# Mechanism of CO_2_ and NH_3_ transport through human aquaporin 1: Evidence for parallel CO_2_ pathways

**DOI:** 10.1113/JP289556

**Published:** 2025-12-04

**Authors:** Raif Musa‐Aziz, R. Ryan Geyer, Seong‐Ki Lee, Fraser J. Moss, Walter F. Boron

**Affiliations:** ^1^ Department of Physiology and Biophysics, Institute of Biomedical Sciences University of São Paulo São Paulo Brazil; ^2^ Department of Physiology and Biophysics Case Western Reserve University School of Medicine Cleveland OH USA; ^3^ Department of Medicine Case Western Reserve University School of Medicine Cleveland OH USA; ^4^ Department of Biochemistry Case Western Reserve University School of Medicine Cleveland OH USA

**Keywords:** CO_2_ permeability, osmotic water permeability, Surface pH, Xenopus laevis oocytes

## Abstract

**Abstract:**

The traditional view had been that dissolved gases cross membranes simply by dissolving in and diffusing through the membrane lipid. However, some membranes are impermeable to CO_2_ and NH_3_, whereas some aquaporin (AQP) water channels—tetramers with hydrophobic central pores—are permeable to CO_2_, NH_3_ or both. Nevertheless, we understand neither the routes that CO_2_ and NH_3_ take through AQP tetramers, nor the basis of CO_2_/NH_3_ selectivity. Here, we show—for human AQP1 (hAQP1)—that virtually all NH_3_ and H_2_O pass through the hydrophilic, monomeric pores. However, CO_2_ passes through both the monomeric pores and another pathway. We expressed hAQP1 in *Xenopus* oocytes and used microelectrodes to monitor the maximal surface‐pH transient (ΔpH_S_) caused by CO_2_ or NH_3_ influxes. We found that p‐chloromercuribenzene sulfonate (pCMBS)—which reacts with C189 in the monomeric pore—eliminates the entire hAQP1‐dependent (*) NH_3_ signal (ΔpH_S_*)_NH3_, but only half of the signals for CO_2_ (ΔpH_S_*)_CO2_ or osmotic water permeability *P*_f_*. 4,4'‐diisothiocyanatostilbene‐2,2'‐disulfonate (DIDS), eliminates the remaining (ΔpH_S_*)_CO2_ but has no effect on (ΔpH_S_*)_NH3_ or *P*_f_*. Together, the two drugs completely eliminate the CO_2_ permeability of hAQP1. When we express hAQP1 in *Pichia pastoris*, treat spheroplasts with DIDS and examine hAQP1 by SDS‐PAGE, reactivity with an anti‐DIDS antibody shows that DIDS crosslinks hAQP1 monomers. Our results provide the first evidence that a molecule can move through an AQP via a route other than the monomeric pore, and raise the possibility that selectivity depends on the extent to which CO_2_/NH_3_ moves through monomeric pores versus an alternate pathway (e.g., the central pore).

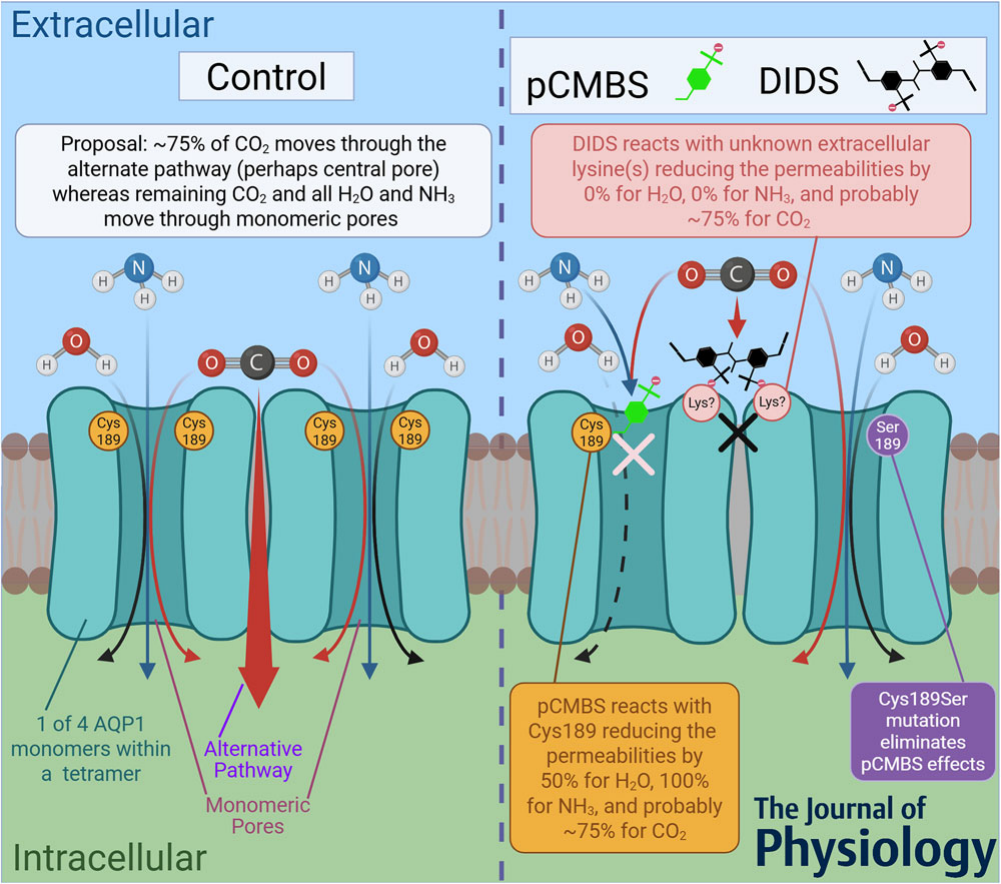

**Key points:**

- Some membranes have negligible CO_2_ permeability in the absence of protein channels like aquaporin‐1 (AQP1).
- We confirm that, during CO_2_ influx, heterologous expression of human AQP1 (hAQP1) in *Xenopus* oocytes increases the magnitude of the transient surface‐pH increase by an amount (ΔpH_S_*)_CO2_, measured with microelectrodes. During NH_3_ influx, hAQP1 expression increases the magnitude of the transient pH_S_ decrease by (ΔpH_S_*)_NH3_.
- p‐chloromercuribenzene sulfonate (pCMBS), which reacts with C189 in the monomeric pore, reduces (ΔpH_S_*)_CO2_ by half; (ΔpH_S_*)_NH3_, to zero; and AQP1‐dependent osmotic water permeability (*P*_f_*), by half.
- 4,4'‐diisothiocyanatostilbene‐2,2'‐disulfonate (DIDS) reduces (ΔpH_S_*)_CO2_ by half, but has no effect on (ΔpH_S_*)_NH3_ or *P*_f_*. DIDS crosslinks AQP1 monomers expressed in *Pichia pastoris*.
- Together, pCMBS+DIDS reduce (ΔpH_S_*)_CO2_ to zero. The C189S mutation of AQP1 eliminates the effects of pCMBS, but not of DIDS. Our results thus show that CO_2_ traverses AQP1 via the monomeric pore plus a novel DIDS‐sensitive route that may be the central pore.

## Graphical abstract

**Abstract figure legend** The figure illustrates the permeation pathways for CO_2_, NH_3_ and H_2_O through AQP1. The panel on the left, with the wild‐type protein under control conditions, CO_2_ moves through monomeric pores (2 of 4 shown) but predominantly via an alternate pathway (perhaps the central pore of the tetramer). The NH_3_ and H_2_O diffuse exclusively via monomeric pores. The panel on the right, with the use of inhibitors and a point mutation, illustrates the dissection of the two distinct pathways that underlie the selectivity of AQP1 for its molecular traffic.

## Introduction

Before the discovery of specific protein‐mediated pathways in cell membranes, investigators had believed that many small molecules (e.g., H_2_O, urea, glycerol, lactic acid)—including CO_2_—freely permeated all membranes to the extent that they can dissolve in and then diffuse through the lipid phase. Solubility‐diffusion theory (S‐DT) embodies this concept (reviewed in Boron, 2010; Michenkova et al., 2021). For molecules other than dissolved gases, the generalisability of this notion faded with the discovery of each new membrane channel or transporter, consistent with the idea that cells evolved to achieve tight control over the transmembrane traffic of small molecules. We now believe that the traffic of such molecules depends on some combination of membrane proteins and S‐DT (responsible for non‐specific leaks).

Regarding CO_2_ traffic across biological membranes, the first inconsistency in the applicability of S‐DT was the demonstration that apical membranes (i.e., facing the lumen) of single gastric glands are impermeable to CO_2_ (Waisbren et al., 1994). The second was the discovery that the human (h) aquaporin‐1 (AQP1), besides its eponymous substrate, H_2_O (Preston & Agre, 1991), also conducts CO_2_ (Nakhoul et al., 1998). Soon after Nakhoul's observation, Cooper and Boron (1998) found that pCMBS inhibits CO_2_ permeability in oocytes expressing wild‐type (WT) hAQP1. Prasad et al. (1998) then reported that AQP1 purified from human red blood cells (RBCs) and reconstituted into lipids from *E. coli* increases CO_2_ permeability, an effect blocked by HgCl_2_. Moreover, Forster et al. (1998) found that 4,4′‐diisothiocyanatostilbene‐2,2′‐disulfonate (DIDS) applied to native human RBCs reduces not only HCO_3_^−^ permeability, but also CO_2_ permeability.

Forster and colleagues recognised that an explanation for their data could be that DIDS blocks a protein pathway for CO_2_ diffusion through the RBC membrane. Indeed, Endeward et al. (2006) later found that AQP1 is responsible for about half of the CO_2_ traffic across the human RBC membrane, with the rhesus (Rh) complex being responsible for most of the rest (Endeward et al., 2008), leaving <10% to be accounted for by S‐DT. Uehlein et al. (2003) reported that NtAQP1 in tobacco plants plays important physiological roles in CO_2_ uptake—driven by a very small air‐to‐chloroplast gradient—for photosynthesis, stomatal opening and leaf growth. Wang and colleagues demonstrated that the CO_2_ permeability of tomato aquaporin PIP2;1 is essential for CO_2_‐dependent regulation of stomatal aperture (Wang et al., 2016).

Besides H_2_O and CO_2_, various AQPs can conduct a variety of substances, including glycerol in the case of members of the aquaglyceroporin subfamily (Gomes et al., 2009). Yasui et al. (1999) found that AQP6, mainly present in intracellular vesicles, has negligible H_2_O permeability but behaves as a non‐selective anion channel. Moreover, a single mutation, N60G, abolishes anion permeability and effectively converts AQP6 to a water channel. Later, Qin and Boron (2013) found that the opposite mutation in hAQP5, G50N, eliminates both H_2_O and CO_2_ permeability, whereas the nearby mutation L51R converts AQP5 into an anion channel blockable by mercury. These residues, although on TM2, are in proximity to the ar/R selectivity filter of the monomeric pore. Yool et al. (1996) reported that, with oocytes expressing hAQP1, forskolin or a cAMP analog increased water permeability and triggered a cation conductance. However, others report that they have not been able to confirm that work (Agre et al., 1997; Deen et al., 1997; Verkman & Yang, 1997).

Regarding dissolved gases, hAQP1 conducts NH_3_ (Nakhoul et al., 2001) and NO (Herrera et al., 2006). Moreover, different AQPs can exhibit strong selectivity for CO_2_ over NH_3_ or vice versa (Geyer et al., 2013; Musa‐Aziz, Chen et al., 2009). More recent work has suggested that *Nicotiana tabacum* PIP1;3 (Zwiazek et al., 2017). Preprint publications have concluded that AQP1, the Rh complex and an unidentified protein are responsible for nearly all O_2_ permeability in murine RBCs (Moss et al., 2025; Occhipinti et al., 2025; Zhao et al., 2025). Moreover, Al‐Samir et al. (2025) report that hAQP1 is permeable to O_2_. These results are consistent with the idea that cells, by regulating channel expression and trafficking to specific membranes, could control the permeability to CO_2_, O_2_ and NH_3_.

Structural studies show that AQP1 is a homotetramer with four independent monomeric pores—lined mainly by hydrophilic residues—that conduct H_2_O (Sui et al., 2001; Walz et al., 1997). At the core of its square‐shaped array of four monomers, AQP1 has a central pore—lined exclusively by hydrophobic residues—with no established function. Each monomer spans the lipid bilayer six times and has cytoplasmic amino and carboxyl termini. As predicted by the ‘hourglass model’ of Preston and Agre (1991), two consensus NPA motifs—on intracellular and extracellular loops that dip in towards the middle of AQP1, near the plane of the membrane—contribute importantly to the monomeric pore. The well‐established reduction of osmotic water permeability (*P*_f_) by HgCl_2_ or pCMBS occurs as these agents react with cysteine‐189 (C189), two residues upstream from the second NPA (Preston et al., 1993). Replacing this cysteine with serine (C189S), which lacks a sulfhydryl group, eliminates this inhibition (Preston et al., 1993).

We previously introduced an approach for using blunt, pH‐sensitive microelectrodes to monitor transient changes in extracellular‐surface pH (pH_S_), caused by the entry of CO_2_ or NH_3_ into *Xenopus* oocytes heterologously expressing membrane proteins (Endeward et al., 2006; Musa‐Aziz, Jiang et al., 2009, Musa‐Aziz, Chen et al., 2010, 2009, 2014a). Mathematical modelling has elucidated the interpretation of CO_2_‐dependent pH transients (Calvetti et al., 2020; Occhipinti et al., 2014; Somersalo et al., 2012). Using this pH approach in a survey of mammalian AQPs, we found that AQPs 1, 6 and 9 are permeable to both CO_2_ (exposure to 5% CO_2_) and NH_3_ (0.5 mM NH_4_Cl); AQP0, the M23 variant of AQP4 (AQP4‐M23) and AQP5 are permeable to CO_2_ but not NH_3_; AQPs 3, 7 and 8 are permeable to NH_3_ but not CO_2_; and AQP2 as well as AQP4‐M1 appear to be permeable to neither (Geyer et al., 2013; Musa‐Aziz, Chen et al., 2009). Using an osmotic‐shrinkage assay with an 80‐fold higher extracellular [NH_3_] than in our studies, Assentoft et al. detected NH_3_ permeability through AQP4‐M23 (Assentoft et al., 2016). A key unanswered question is how various AQPs exhibit such strikingly different CO_2_/NH_3_ selectivities. The answer presumably lies in the pathways that these solutes take through various AQP tetramers. However, despite insights from molecular‐dynamics simulations, we still lack physiological evidence that establishes the pathways by which either CO_2_ (Hub & de Groot, 2006; Wang et al., 2007) or NH_3_ (Kirscht et al., 2016) moves through any AQP. In the case of AtTIP2;1 from the plant *Arabidopsis thaliana*, the crystal structure reveals a unique selectivity filter with a side pore. Kirscht et al. (2016) suggest that NH_4_^+^ could enter from the vacuolar surface (topologically equivalent to the extracellular side), followed by deprotonation to NH_3_+H^+^. The NH_3_ would move along the monomeric pore, whereas the H^+^ would recycle via the side pore to the vacuolar space.

The purpose of the present study is to explore the pathway(s) by which CO_2_ and NH_3_ diffuse through human AQP1. Our approach was to use a combination of cell physiology and biochemistry. Heterologously expressing hAQP1 or its C189S mutant in *Xenopus* oocytes, and using the pH_S_ approach, we assessed the effects of pCMBS and DIDS on CO_2_ and NH_3_ permeation. We also assessed *P*_f_ so that we could normalise CO_2_ and NH_3_ data to H_2_O permeability. We examine pCMBS because mercury—acting on monomeric pores—reduces both the *P*_f_ (Preston et al., 1993) and CO_2_ permeability of hAQP1 (Cooper & Boron, 1998), as expressed heterologously in oocytes. We work with DIDS because this drug reduces CO_2_ permeability in RBCs and in oocytes expressing hAQP1 (Endeward et al., 2006). We find that virtually all of the hydrophilic NH_3_ molecules—as well as H_2_O, as expected—pass through the four hAQP1 monomeric pores, which each possess three hydrophilic nodes in their selectivity filters that are located at the centre of an otherwise long hydrophobic channel (Sui et al., 2001). On the other hand, the more hydrophobic CO_2_ travels both via this pCMBS‐sensitive pathway—the only established pathway through any AQP— and as an independent pathway that we can block with DIDS. Studies on hAQP1 expressed in *Pichia pastoris* indicate that DIDS crosslinks hAQP1 monomers. Our work is the first to demonstrate that a substance can move through an AQP via a pathway—possibly the hydrophobic central pore—that is distinct from the monomeric pore. Considering that mutations in human AQPs are associated with a wide variety of pathological conditions (see Verkman, 2008; Verkman et al., 2008), our work could lead to new insights into disease mechanisms, diagnostic tools and therapeutic approaches for improving clinical outcomes.

## Methods

For previous summaries of our approach in oocyte experiments, see Musa‐Aziz et al. (2010), Musa‐Aziz, Chen et al. (2009), Musa‐Aziz, Jiang et al. (2009). The following description is in greater depth.

### Ethical approval and animal procedures

The Institutional Animal Care and Use Committee at Case Western Reserve University approved the protocols for housing and handling of *Xenopus laevis*, used as a source of oocytes, and rabbits for generating polyclonal antibodies [PHS Assurance number ‐ D16‐00089 (A3145‐01)].

#### Frogs

We purchased adult female *Xenopus laevis* frogs (NASCO Inc., Fort Atkinson, WI, USA) and housed them in a 20‐gallon static aquarium, managed by the Animal Resources Centre (ARC) of the School of Medicine. For stress mitigation, the tank had six or fewer frogs, and for environmental enrichment, we included a PVC elbow pipe. A charcoal Bio‐Bag aquarium power pump (Tetra, Blacksburg, VA) circulated dechlorinated water through the tank. Three times per week, ARC staff fed the frogs with adult *Xenopus* diet (Zeigler Bros. Inc., Gardners, PA), sprinkling the food (10 pellets/frog) into the tank and, after a few hours when the *Xenopus* had fed, removing excess food with a net. As judged necessary, the ARC staff partially changed out the water in the tank. Every 90 days, they moved the frogs into a newly cleaned tank that contained, in equal amounts, water from the previous tank and new de chlorinated water.

We anaesthetised frogs by immersion in a solution containing 0.2% tricaine (i.e., MS‐222; ethyl 3‐aminobenzoate methanesulfonate, catalogue # A5040, Sigma‐Aldrich, St Louis, MO, USA). When an animal became unresponsive to touch, we removed it from the solution and surgically extracted the ovaries. The animal was euthanized by cardiac excision prior to recovery from anaesthesia.

In some experiments, we isolated oocytes from *Xenopus* ovarian lobes shipped overnight from NASCO.

#### Rabbits

We purchased adult female New Zealand white rabbits—named ‘Ren’ and ‘Stimpy’ for internal identification of the source of antiserum—from Charles River Laboratories (Ashland, OH). The rabbits were housed individually in stainless‐steel cages in a temperature‐controlled room (20–22°C) with a 12 h light/12 h dark cycle with free access to commercially available pelleted rabbit chow and fresh water. They were observed daily by trained animal care personnel in the Animal Research Centre (ARC) to monitor general health and well‐being. Following IACUC guidelines, ARC staff obtained a 5‐mL pre‐immune blood sample from an ear vein and then inoculated two rabbits—gently restrained in a rabbit‐restraint cage and lightly sedated with intramuscular acepromazine (2 mg/kg)—with fresh DIDS‐KLH fusion protein (see below^1^) and Freund's complete adjuvant via subcutaneous injection on the back of the rabbit. A 10‐mL sample of blood (via ear veins, alternating sides) was taken 14–21 days after the first boost, and the sera were evaluated for antigenicity towards proteins labelled with DIDS. Once a rabbit began producing sufficient titers of polyclonal anti‐DIDS antibodies, staff collected 10 mL of blood per kg of body weight (∼30–50 mL total) at intervals of 21 to 30 days. After two such larger blood collections, ARC staff euthanized the animals by cardiac excision and exsanguination, with blood from the terminal collection being transferred to our laboratory.

### Solutions and chemicals

#### OR3 media (for maintaining oocytes)

We follow the protocol described in (Musa‐Aziz et al., 2010). Briefly, we added to 1.8 L of H_2_O one pack of powdered Leibovitz L‐15 media with L‐glutamine (catalogue # L4386, Sigma–Aldrich), 100 mL of 10,000 U/mL penicillin/streptomycin solution (cat # 15 140 122, Thermo Fisher Scientific, Waltham, MA, USA; hereafter abbreviated TFS), and 5 mM HEPES free acid. We then titrated the solution to pH 7.50 with NaOH, periodically measuring an osmolality of 195 mOsm, sterile‐filtered the solution using a Corning Disposable Vacuum Filter/Storage system (catalogue # 09‐761‐107, TFS), and stored it in a cold room for up to 2 weeks before use. Finally, just before use, we sterile‐filtered a small volume of solution using a 60‐mL syringe (catalogue # 14‐955‐461, TFS) and a sterile in‐line Nalgene filter with a 0.22‐µm pore size and a 25‐mm diameter (catalogue # 723‐9920, TFS).

#### Solutions for physiology experiments

Table 1 describes the final composition of nine solutions used in the physiology experiments. All were assembled and used at room temperature (∼22°C). Where required, we describe any necessary additional steps for correct solution assembly (e.g., the order in which components should be added) below.

**Table 1:** Solutions^*^

	1	2	3	4	5	6	7	8	9
Solution Component or parameter	ND96	Ca^2+^‐free ND96	5% CO_2_/ 33 mM HCO_3_^−^ [for (ΔpH_S_)_CO2_]	5 mM NH_3_/NH_4_^+^ in ND96	0.5 mM NH_3_/NH_4_^+^ in ND96 [for (ΔpH_S_)_NH3_]	Hypotonic ND96 [for *P*_f_]	ND96+ pCMBS	ND96+ DIDS	BSA [to scavenge DIDS]
NaCl (mM)	96	98.7	63	91	91	33	96	96	96
KCl (mM)	2.0	2.0	2.0	2.0	2.0	2.0	2.0	2.0	2.0
CaCl_2_ (mM)	1.8	0	1.8	1.8	1.8	1.8	1.8	1.8	1.8
MgCl_2_ (mM)	1.0	1.0	1.0	1.0	1.0	1.0	1.0	1.0	1.0
HEPES (mM)	5	5	5	5	5	5	5	5	5
NaHCO_3_ (mM)	0	0	33	0	0	0	0	0	0
NH_4_Cl (mM)	0	0	0	5	0.5	0	0	0	0
CO_2_ (%)	0	0	5	0	0	0	0	0	0
pH (adjusted with NaOH)	∼7.50	∼7.50	∼7.50	∼7.50	∼7.50	∼7.50	∼7.50	∼7.50	∼7.50
Temperature (°C)	RT	RT	RT	RT	RT	RT	RT	RT	RT
Osmolality (mOsm)	∼195	∼195	∼195	∼195	∼195	∼80	∼195	∼195	∼195
pCMBS (mM)	0	0	0	0	0	0	1	0	0
DIDS (µM)	0	0	0	0	0	0	0	100	0
BSA %	0	0	0	0	0	0	0	0	0.2%

* This table shows the compositions of all the solutions used in the physiology experiments the present paper.

Abbreviations: BSA, Bovine serum albumin; DIDS, 4,4'‐diisothiocyanatostilbene‐2,2'‐disulfonate; pCMBS, p‐chloromercuribenzene sulfonate, *P*_f_, osmotic water permeability.

#### (Solution 3) 5% CO_2_/33 mM HCO_3_^−^ for measuring ΔpH_S_ due CO_2_ influx

After adding all components except NaHCO_3_, we titrated the solution to pH 7.50, then added 33 mM NaHCO_3_, and then equilibrated the solution with 5% CO_2_ (balanced with air), which brought pH back to 7.50.

#### (Solutions 4 and 5) 0.5 mM NH_3_/NH_4_^+^ in ND96 for measuring ΔpH_S_ due NH_3_ influx

We first made the 5 mM NH_3_/NH_4_^+^ in ND96 solution (Solution #4) in which we replaced 5.0 mM NaCl with an equivalent amount of NH_4_Cl, and then diluted this solution 1:10 into ND96 to create the final 0.5 mM NH_3_/NH_4_^+^ solution (Solution #5).

#### (Solution 6) Hypotonic ND96 for measuring P_f_

This is a variant of ND96 in which we reduced [NaCl] to lower osmolality to 80 mOsm/kg.

#### (Solution 7) ND96+pCMBS

In some experiments, we pre‐incubated oocytes for 30 min in ND96 containing 1 mM pCMBS (catalogue # C367750, Toronto 196 Research Chemicals, North York, Ontario, Canada), a sulfhydryl reagent, added to the solution as dry powder to ND96 (Solution #1).

#### (Solution 8) DIDS

In some experiments, we pre‐incubated oocytes for 1 h in an ND96 containing 100 µM DIDS (catalogue # D3514, Sigma–Aldrich), an amino‐reactive agent added to the solution as a dry powder to ND96 (Solution #1).

#### (Solution 9) BSA (Bovine serum albumin, to scavenge DIDS)

In some experiments, after DIDS pretreatment, we washed off unreacted DIDS by exposing oocytes to an ND96 to which we added 0.2% BSA (catalogue # A9418, Sigma–Aldrich) directly to ND96 (Solution #1).

When assembling all solutions, we measured pH using a Ross electrode (catalogue # 927007MD, TFS), connected to a Dual Star pH meter (catalogue # 8102BNUWP, TFS) and calibrated with two standards (from TFS), buffer solution standards pH 6.0 (catalogue # SB104‐1) and pH 8.0 (catalogue # SB112‐20). We measured osmolality with a vapor‐pressure osmometer (catalogue # 5520 Vapro, Wescor Inc., Logan, UT).

### Oocyte isolation

We placed ovarian lobes in a sterile 100‐mm Petri dish containing ND96, cut them into irregular pieces (<1 cm in size) containing ∼10 oocytes each using small iridectomy scissors. We then poured the mixture into a new Petri dish containing ∼15 mL of Ca^2+^‐free ND96 and gently agitated the dish on a horizontal shaker^2^ for 10 min, poured off as much liquid as possible, added fresh Ca^2+^‐free ND96, and repeated the pour/shake/wash cycle twice more. We then poured off the Ca^2+^‐free ND96 before adding ∼15 mL of a freshly‐made mixture of Ca^2+^‐free ND96 and 2 mg/mL Collagenase Type IA (catalogue # **C9722**, Sigma–Aldrich), and gently agitating the dish on a horizontal shaker for 40 min. We next poured off the collagenase solution, added collagenase‐free Ca^2+^‐free ND96, gently agitated the dish for 15 min, and repeated the pour/shake/wash cycle twice more. We then poured off the Ca^2+^‐free ND96, added ND96 (i.e., containing Ca^2+^), gently agitated the dish on a horizontal shaker for 15 min, and repeated the pour/shake/wash cycle twice more. Finally, we poured off the ND96, added ∼15 mL OR3 media, repeated the pour/wash cycle twice more, poured off most of the liquid, transferred the oocytes and remnant OR3 media to a fresh Petri dish containing fresh OR3 media, and incubated the oocytes in an incubator at 18°C. Later on the same day, we used a stereomicroscope to sort the oocytes and select individual, defolliculated, mature oocytes (stage V/VI), which we immediately moved to a 6‐well plate containing OR3 media (as many as ∼50 oocytes/well), using a fire‐sterilised transfer pipette prepared by using a diamond pencil to score the tapered end of a Pasteur pipette at a diameter large enough to accommodate an oocyte, breaking off and discarding the thin end of the pipette, fire‐polishing the cut end of the pipette, and attaching the large end of the pipette to a manual 2‐mL Pipette Pump (catalogue # S3‐594‐3, TFS). We incubated the oocytes overnight at 18°C before injecting them the following day with *cRNA* or H_2_O (see below).

### cRNA synthesis

The cDNAs encoding both hAQP1‐WT (accession# NM_198 098) and hAQP1‐C189S were gifts of Dr. Peter Agre (Johns Hopkins University). We subcloned the open reading frames of the constructs into the expression plasmid pGH19 (Trudeau et al., 1995), a vector containing the 3′ and 5′ untranslated regions of the *Xenopus* laevis β‐globin gene. In experiments in which we expressed hAQP1‐WT or hAQP1‐C189S in *P. pastoris*—as well as in some control experiments in oocytes—we added an N‐terminal (Nt) FLAG‐tag (MASEFKKKL…; FLAG sequence, underscored; hAQP1 sequence, double underscored).

We linearised cDNA constructs using NotI (for untagged hAQP1‐WT and hAQP1‐C189S) or XhoI (for the hAQP1‐FLAG tagged versions). The linearised DNA was then purified using the QIAquick PCR purification kit (catalogue # 28 104, Qiagen Inc., Valencia, CA). We then synthesised capped RNA (cRNA) using T3 (for untagged constructs; catalogue # AM1348, Ambion, Austin, TX, USA ) or T7 (for FLAG‐tagged constructs; catalog # AM1344, Ambion) mMessage mMachine kits (Ambion, Austin, TX, USA). The cRNA was purified using the RNeasy MinElute RNA Cleanup Kit (catalogue # 74 204, Qiagen). The cRNA concentration was determined based on ultraviolet absorbance at 260 nm, and quality was assessed according to the A260/280 ratio and gel electrophoresis.

### Injection of oocytes with cRNA or water

After isolation, defolliculation and sorting (Day 0), we injected stage V–VI oocytes with 25 nL of cRNA (1 ng/nL) encoding hAQP1‐WT, its C189S mutant, or the FLAG‐tagged versions, or ∼25 nL of water as a control (Day 1). The injection apparatus consisted of a Nanoject II Variable Volume Automatic Injector (Drummond Scientific Company, Broomall, PA) connected to an injection needle that we pulled from 10‐µL microdispenser capillary glass tubing (catalogue # 3‐000‐210‐G, Drummond), using a Model P‐97 Flaming/Brown Micropipette Puller (Sutter Instrument Company, Novato, CA) and then modified by manually breaking the sealed end to yield a tip diameter of ∼50 µm. After injection, we transferred oocytes to a 6‐well plate (up to ∼50/well) and maintained them in OR3 media and maintained them for 3 days in an incubator at 18°C before use in experiments (generally on Day 4).

### Electrophysiological recordings

For a schematic representation of the experimental chamber, position of the oocyte and arrangement of electrodes, see Fig. 8*B* in Musa‐Aziz et al. (2010).

#### Chamber and solution delivery

We placed oocytes into the channel (3 mm wide × 2 mm deep × 30 mm long) of a plastic perfusion chamber. We delivered the physiological solutions—or pH standards for calibrating pH microelectrodes at the outset of an experiment (see next section)—at a constant, total flow of 4 mL/min at room temperature (∼22°C), using a series of dual‐syringe pumps (Model 22, Harvard Apparatus, South Natick, MA), each of which drove two 140‐mL plastic syringes (Sherwood Medical, St. Louis, MO) of identical contents to deliver two solutions at 2 mL/min each.^3^ The two solutions from each pump flowed through separate lengths of Tygon tubing (5/32‐inch (∼4.0 mm) outer diameter, 3/32‐inch (∼2.4 mm) inner diameter; Ryan Herco Products Corp., Burbank, CA; Formulation R3603‐3; OD 4.8 mm/ID 1.6 mm to two parallel assemblies of pneumatically operated 5‐way valves (Clippard Instrument Laboratory, Cincinnati, OH), daisy‐chained in such a way that we could switch amongst identical pairs of solution lines. The output of the parallel switching assemblies flowed into two lengths of Tygon tubing that carried the solutions to the vicinity of the chamber, where the two lines merged in a ‘mixing T’, the output of which flowed into one end of the chamber channel. A suction device removed the solution at the opposite end of the channel. See Musa‐Aziz et al. (2014a, 2014b) for details.

#### Measurement of membrane potential and intracellular pH

As described previously (Musa‐Aziz et al., 2010, 2014a), we impaled the oocyte (with the dark animal pole facing upward) with two microelectrodes, one for measuring membrane potential and the other for measuring intracellular pH. Both microelectrodes, with tip diameters of ∼1 µm, we pulled from thin‐walled borosilicate microfilament glass tubing (catalogue # G200TF‐4, 2.0 mm OD ×1.56 mm ID, Warner Instruments Corporation, Hamden, CT), using the aforementioned P‐97 microelectrode puller.

The microelectrodes for measuring membrane potential (*V*_m_) were filled with 3M KCl. We inserted an electrode into a half‐cell/microelectrode holder (model # ESW‐F20N, Warner Instruments Corp.), attached the holder to the *V*_m_ probe of an OC‐725 two‐electrode Oocyte Clamp (Warner Instruments Corp.), and mounted the probe on a model MM‐33R micromanipulator (Warner Instruments Corp.). The *V*_m_ microelectrodes had resistances of ∼0.3–∼0.6 MΩ.

The microelectrodes for measuring intracellular pH (pH_i_) were identical to the *V*_m_ electrodes except for the following fabrication process. In order to remove moisture, we placed the pulled micropipettes, with tips upwards, into the cylindrical holes of a reusable aluminum block (machined to create ‘legs’ and thereby allow gases to circulate from beneath, and with smaller concentric cylindrical holes extending the entire height of the block to allow access of gases from below, to the interior of the glass capillary), placed the block into the bottom of a 100‐mm Pyrex petri dish, covered the electrodes and block with an inverted 250‐mL beaker, placed the petri dish on an open metal rack of an oven at 200 °C, and then baked overnight. With the microelectrodes still in the 200 °C oven, we then silanized them by tilting the beaker slightly and depositing 80 µL of *bis*‐di‐(methylamino)‐dimethylsilane (Sigma–Aldrich, catalogue #14 755) between the ‘legs’ of the aluminum block, releasing the beaker, and allowing the silane fumes to interact with the microelectrodes for 40 min before removing the beaker. The silanized electrodes were cured in the oven at 200 °C until ready for use. We used a hand‐drawn, soft‐glass pipette with a long tip to backfill each pH_i_ electrode with a liquid pH‐sensitive sensor (catalogue # 95 293, Hydrogen Ionophore I, mixture B, Fluka Chemical Corp., Ronkonkoma, NY), as described by Ammann et al., 1981, creating a column that extended ∼1 mm from the microelectrode tip. We then backfilled a microelectrode with a buffer solution (containing, in mM, 40 KH_2_PO_4_, 23 NaOH, 15 NaCl, adjusted to pH 7.0), inserted the electrode into a half‐cell/microelectrode holder (model # ESW‐F20N, Warner Instruments Corporation), attached the holder to one probe of a model FD223 dual high‐impedance electrometer, World Precision Instruments (WPI), Inc., Sarasota, FL), and mounted the probe on a model MM‐33R micromanipulator (Warner Instruments Corp.). The pH_i_ microelectrodes had resistances of ∼0.3–0.6 MΩ.

After placing the tips of the *V*_m_ and pH_i_ microelectrodes in ND96 solution in the chamber channel (which we now refer to as the ‘bath’), we obtained *V*_m_ by an analogue electronic subtraction of the system ground of the OC‐725 amplifier from the signal of the *V*_m_ electrode. We similarly obtained the voltage due to pH_i_ by electronically subtracting the signal of the *V*_m_ electrode from the signal of the pH_i_ electrode. For a schematic representation of the configuration of the electronics for obtaining *V*_m_ (this section) and the voltages due to pH_i_ (this section) and pH_S_ (next section), see Fig. 7*C* in Musa‐Aziz et al. (2010). The device that performed the subtractions (Yale University Subtraction Amplifier, v3.1) also appropriately scaled the voltages for the inputs of an analogue‐to‐digital converter, installed in a Windows‐based computer. With the tips of the *V*_m_ and pH_i_ electrodes (and also the pH_S_ electrode, simultaneously; see next section) in the bath, we obtained the slope of the pH_i_ electrodes as we used the aforementioned solution‐delivery system (see previous section) to flow a pH standard at pH 6.0 (catalogue # SB104‐1) continuously through the chamber channel, recorded the voltage due to that pH, used the valve system to switch the flowing solution to a second pH‐8.0 standard (catalogue # SB112‐20), and then recorded the corresponding voltage. We typically switched between standards in a pH 6 → 8 → 6 → 8 sequence, accepting pH_i_ microelectrodes that completed their electrical response—which also included the time for the solution in the chamber channel to fully change composition—within ∼5 s (∼2 s for pH_S_ electrodes) and had slopes in the range of 53 to 60 mV/pH unit. After the recordings in the final pH‐calibration solution, we reintroduced the ND96 solution into the bath and then added the oocyte to the chamber channel. After impaling the oocyte with the *V*_m_ electrode, and with the tip of the pH_i_ electrode near the oocyte, we obtained a single‐point pH calibration of the pH_i_ microelectrode in the ND96 solution (defined as having a pH of 7.50). We then impaled the oocyte with the pH_i_ microelectrode. After stabilisation, oocytes had spontaneous *V*_m_ values at least as negative as –40 mV. We continuously recorded pH_i_ to judge the integrity of the oocyte and compare the pH_i_ time course with that of surface pH. In the present paper, we are not presenting the pH_i_ data, which nevertheless are similar to those of our previous studies (Musa‐Aziz, Chen et al., 2009; Musa‐Aziz et al., 2010; 2014a, 2014b).

#### Measurement of surface pH

We measured surface pH with liquid‐sensor pH microelectrodes that we fabricated and employed in the same way as we did for pH_i_ microelectrodes (see above), but with four differences. First, we pulled the pH_S_ microelectrodes from standard‐walled (rather than thin‐walled) borosilicate microfilament glass tubing (Part No. G200F‐4, 2.0 mm OD ×1.16 mm ID, Warner Instruments). Second, we used a microforge to break off and fire‐polish the tips (inner diameter ≅ 20 µm at tip) as one would for a giant‐patch pipette (Musa‐Aziz et al., 2010). It was to facilitate the fire polishing that we used the standard‐walled glass tubing. Third, we used a vented microelectrode holder (model # ESW‐F20N, Warner Instruments Corp.) mounted on a model MM‐33L micromanipulator. The vent prevented pressure buildup that would otherwise have pushed the liquid pH sensor out of the wide electrode tip as we pushed the pH_S_ electrode into the holder. Note that we attached the microelectrode holder of the pH_S_ electrode to the second of two inputs of the FD223 electrometer. And fourth, after obtaining the pH_S_‐electrode slope (which we did at the same time as obtaining the pH_i_ electrode slope, as described above) and single‐point calibration of the pH_S_ electrode in ND96, we used an ultra‐fine computer‐controlled micromanipulator (model MPC‐200 system, Sutter Instrument Company) to position the blunt tip of the pH_S_ electrode near the oocyte's equator (i.e., between the upward‐facing animal pole and the downward‐facing vegetal pole), and ∼5 degrees behind the meridian (i.e., barely in the ‘shadow’ of the flowing extracellular solution) until the pH_S_ electrode tip just touched the surface of the oocyte (Musa‐Aziz et al., 2010, 2014a). We then further advanced the tip by ∼40 µm to create a slight dimple in the membrane. Periodically during the experiment (indicated by vertical grey bands in each panel of Figs 2*B*, 3 and 6), we withdrew the electrode ∼300 µm from the oocyte for recalibration in the bulk extracellular fluid (bECF) of the bath (i.e., pH 7.50).

The external reference electrode for the pH_S_ measurement was a calomel half‐cell, bridged to the chamber channel via a long glass micropipette filled with 3M KCl, connected to a model 750 electrometer (WPI), and positioned so that its tip was just downstream of the oocyte. We obtained the pH_S_ signal by an analogue electronic subtraction of the calomel signal from the signal of the pH_S_ electrode.

We established a virtual ground with an Ag/AgCl half cell (connected to the *I*_Sense_ input of the voltage‐clamp amplifier) bridged to the chamber by a second glass microelectrode filled with 3M KCl, and positioned the tip of this second bridging pipette close to that of the first, that is, just downstream of the oocyte.

#### Measurement of osmotic water permeability

We measured *P*_f_ as described by Preston et al. (1992, 1993) and Zhang et al. (1990). Using an approach similar to those in previous studies by our group (Musa‐Aziz, Chen et al., 2009; Virkki et al., 2001), we dropped an oocyte into a Petri dish containing a hypotonic solution (80 mOsm; Solution #6, Table 1) to induce cell swelling and, whilst illuminating from beneath the dish, used a video camera to obtain images of the oocyte (1/s × 60 s). Using a small metal sphere next to the oocyte as a size reference, we determined the projection areas of the oocyte, and—assuming the oocyte to be a perfect sphere—computed idealised oocyte volume (V_Oocyte_) and idealised oocyte surface area as a function of time. We estimated the actual surface area (*S*, in cm^2^) by multiplying the idealised surface area by a factor of 9 (Chandy et al., 1997). We computed *P*_f_ from the following equation:

$$P_{f}=\underset{\mathrm{units}:\;cm/s}{\underset{︸}{\;\frac{\;{\left(\frac{dV_{\mathrm{Oocyte}}}{dt}\right)}_{\max}}{S\times \Delta Osm\times V_{W}}}}\;,$$

the form of which differs slightly from that of Zhang et al. (1990).^4^ Here, (*d*V_Oocyte_/*dt*)_max_ is the maximum time rate of change of cell volume (i.e., the maximal H_2_O influx, or *J*_V,max_, in cm^3^ s−^1^), Δ*Osm* is the initial osmotic gradient across the oocyte membrane (i.e., 195–80=115 mOsm, expressed as mol cm−^3^), and V_w_ is the molar volume of water (18.1 cm^3^ mol−^1^). As for the electrophysiological studies, we performed all *P*_f_ assays at room temperature (∼22°C).

### Protocols for physiological experiments

**Figure 1:**
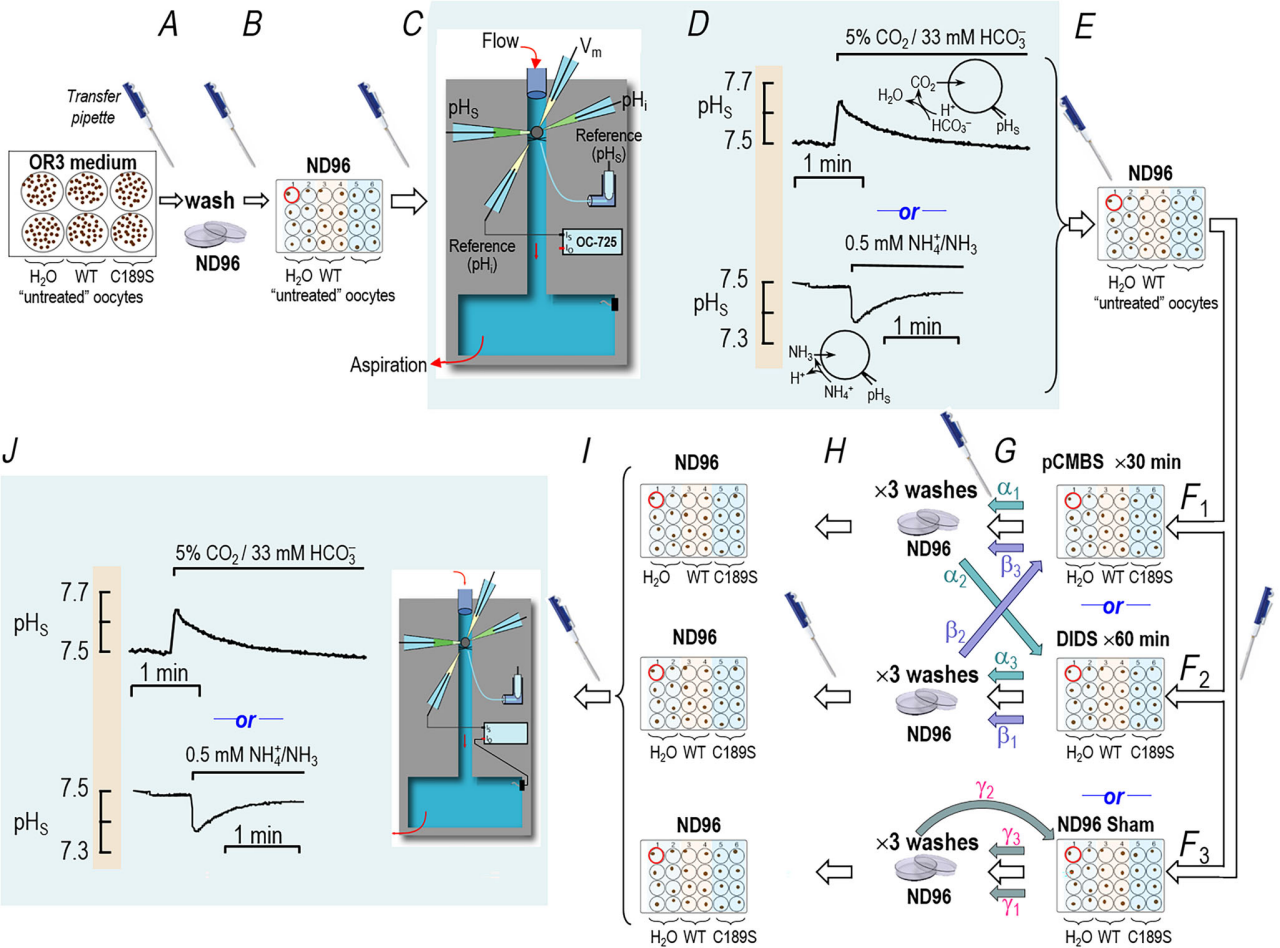
Flow chart showing experimental protocols The letters ‘A’, ‘B’, ‘C’ and so on are above broad arrows and near icons of a pipettor that identify the transfer of an oocyte (using a transfer pipette) from one step in our protocol to another. ‘D’ and ‘J’ are the initial and final electrophysiological recordings, respectively. The oocytes may be controls (injected with H_2_O) or those expressing either hAQP1‐WT (AQP1) or hAQP1‐C189S (C189S). In transfer ‘F’, we move the oocyte to a solution containing pCMBS (F_1_), DIDS (F_2_), or no drug (F_3_). As indicated by the white arrows, we can directly transfer from F_1_ (or F_2_ or F_3_) to G to H. Alternatively, at transfer ‘F’, we can execute subsidiary protocols (α/teal arrows, β/lavender arrows, γ/grey arrows) in which we return oocytes from washes in ND96 back to a solution containing an inhibitor (i.e., pCMBS or DIDS) or ND96 (i.e., sham drug exposure). Thus, an oocyte might undergo the teal‐colored pCMBS/DIDS sequence (α_1_→α_2→_α_3_), the lavender‐colored DIDS/pCMBS sequence (β_1_→β_2→_β_3_), or the grey‐colored sham/sham sequence (γ_1_→γ_2→_γ_3_). After the transfer H, we perform the final electrophysiology recordings.

Fig. 1*A*–*J* illustrates the sequence of events in our experimental protocols.

#### Assessing oocyte health

Because hAQP1, with its encoding cRNA injected as described above, tends to stress the oocytes, we generally performed experiments on Day 4. We judged the health of the oocytes by microscopic observation of shape and colour, by *V*_m_ (more negative than –40 mV, as described above), and by a firm membrane as we pushed the pH_S_ electrode up against the surface (Wang et al., 2025).

#### Electrophysiological assays

**Figure 2:**
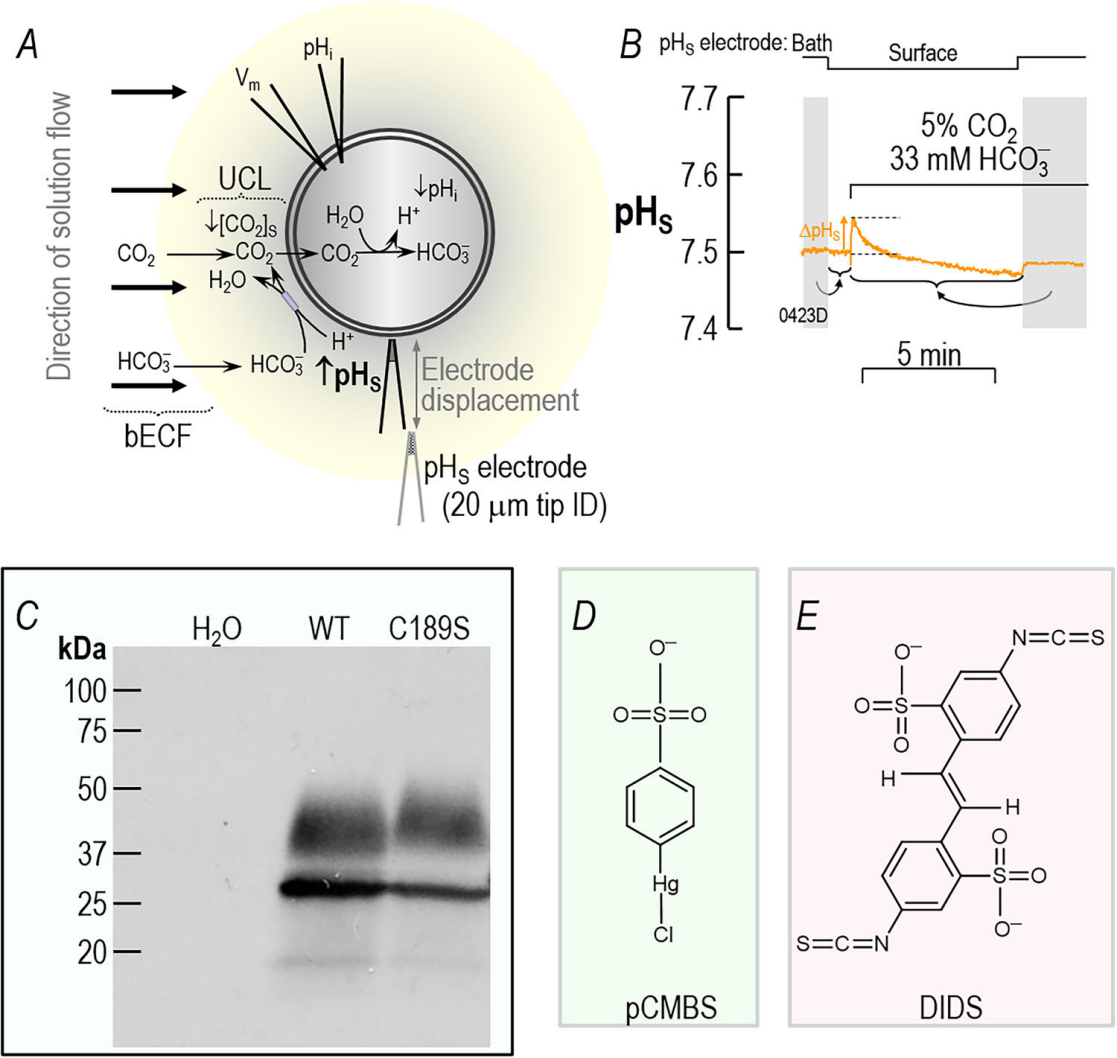
CO_2_ influx into an oocyte. A, schematic illustration of a CO_2_‐influx experiment. Thick black arrows indicate the direction of convective flow within the bulk extracellular fluid (bECF). Thinner arrows indicate solute diffusion or reactions. The maize‐colored halo indicates the layer of extracellular unconvected fluid (EUF). At the outer surface of the membrane, CO_2_ influx creates a CO_2_ deficit, in part replenished by the reaction HCO_3_^−^+H^+^→CO_2_+H_2_O (which raises pH_S_) and in part replenished by diffusion from the bECF (an isohydric process). The double‐headed arrow indicates displacement of the pH_S_ electrode from the cell surface to the bECF for recalibration. ID, inner diameter; UCL, unconvected layer. B, sample surface‐pH (pH_S_ record). The upward arrow indicates the maximal change in pH_S_ (ΔpH_S_) during the application of CO_2_/HCO_3_^−^. During the two periods indicated by the vertical grey bands, the tip of the pH_S_ electrode was displaced ∼300 µm away from the cell surface for recalibration in the bulk extracellular fluid (i.e., pH = 7.50). The curved grey arrow points to horizontal braces that indicate the portions of the experiment to which each calibration pertains. The filename for this representative trace, ‘0423D’—that we reuse in Fig. 3*A*—is annotated at the bottomleft corner of the panel. C, western blot, probed with an anti‐AQP1 antibody, showing relative expression of hAQP1‐WT versus hAQP1‐C189S in one of 3 membrane preparations of oocytes, each from a different donor frog and different cRNA injections. For each blot, we used the ImageJ gel‐analyzer plug‐in to group bands at all molecular weights in each lane to quantify aggregate band intensities. For each blot, we normalised the hAQP1‐WT intensity to 100%, and expressed hAQP1‐C189S intensity as a fraction of hAQP1‐WT. The analysis showed that hAQP1‐C189S expression in the 3 blots was 72.5%, 59.8% and 117.7% of WT; thus, the average was 83.3%±30.4% of WT (mean±SD). A Student's *t*‐test with Welch's correction yielded a *P*‐value of 0.44, indicating no significant difference between hAQP1‐WT versus hAQP1‐C189S mutant expression levels. D, structural formula of pCMBS. E, structural formula of DIDS.

**Figure 3:**
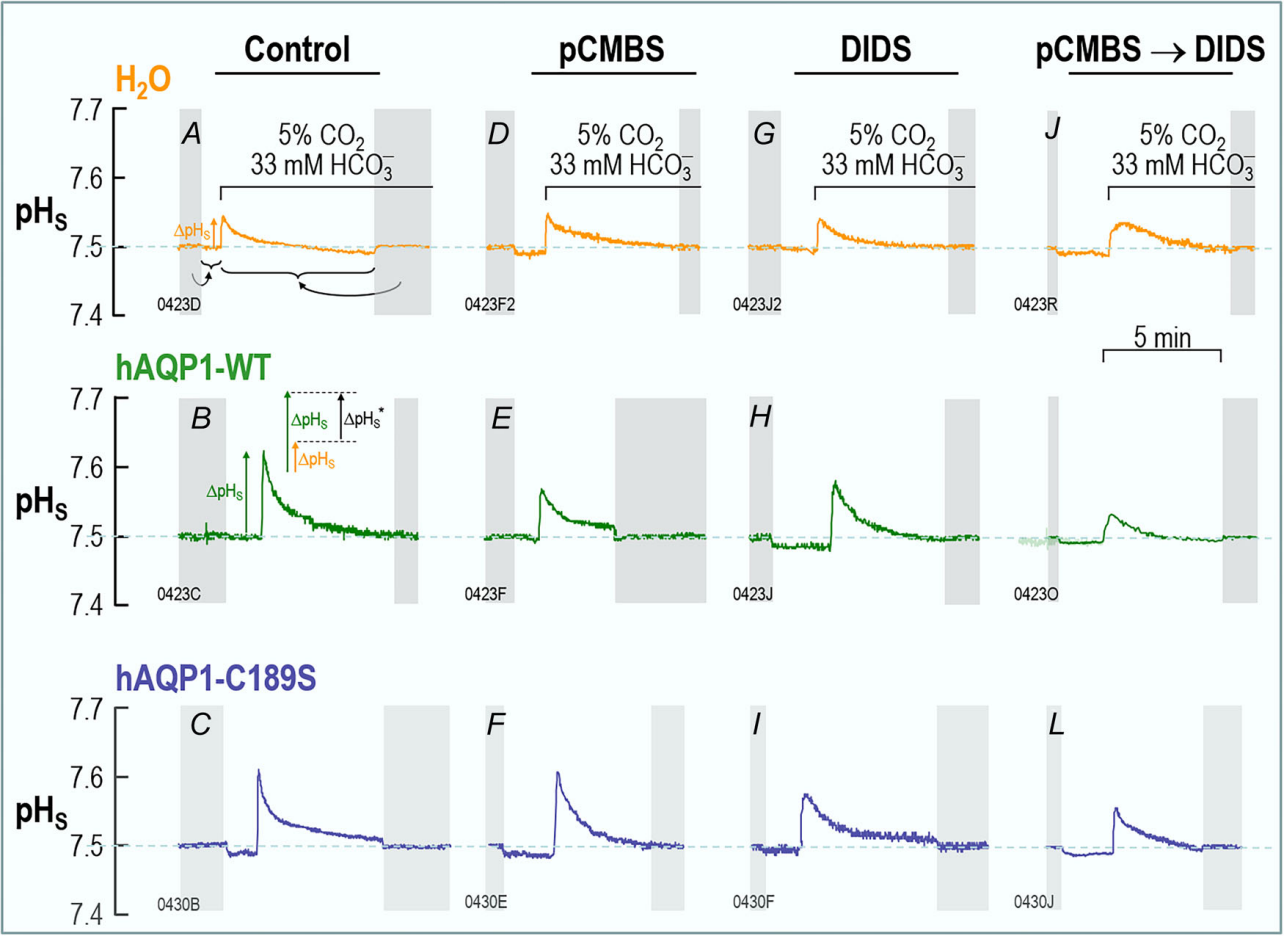
Surface‐pH transients triggered by a CO_2_/HCO_3_^−^ exposure and subsequent CO_2_ influx The figure shows representative pH_S_ records from oocytes injected with H_2_O (top row) or cRNA encoding hAQP1‐WT (middle row) or its C189S mutant (bottom row), and later exposed first to the ND96 solution and then, at the indicated times, to a solution containing CO_2_/HCO_3_^−^. All oocytes underwent the ‘Control’ protocol, followed by one or two of the three indicated F_1_/F_2_/F_3_ protocols in Fig. 1, although some oocytes did not survive beyond the control protocol. As necessary, we pre‐incubated oocytes in 1 mM pCMBS for 30 min or in 100 µM DIDS for 1 h. Neither drug was present in the bulk solution at the time of the assays. The two grey bars in each panel indicate when we withdrew the pH‐electrode tip from the oocyte surface to the bulk extracellular fluid (pH 7.50) for recalibration. In panel A, the curved grey arrows point and horizontal braces have the same meanings as in Fig. 2*B*, and the upward gold arrow indicates the control ΔpH_S_. In panel B, the upward green arrow indicates the ΔpH_S_ in an oocyte expressing hAQP1. Statistics Table 3 presents the analysis of the mean ΔpH_S_ magnitude recorded from all H_2_O, hAQP1‐WT or hAQP1‐C189S ‘control’ oocytes for which we show representative traces in panels *A*, *B* and *C*. The inset shows that the difference in heights of the green arrow and the gold arrow (its day‐matched control) is the channel‐dependent ΔpH_S_ (ΔpH_S_*).

On the day of an experiment, we followed a 10‐step protocol:

- Use a transfer pipette to pick up an untreated oocyte from a well of a 6‐well plate (each well containing OR3 media + ∼30 oocytes, all injected with the same material) and drop the oocyte into a Petri dish containing ND96 (Solution #1, Table 1). After flushing the transfer pipette tip several times with ND96 (to remove OR3).
- Use the transfer pipette to move the washed oocyte to an identified well of a 24‐well plate (1 oocyte/well) containing ND96. Repeat this procedure for several oocytes, each of which will remain in its well (1–3 h) until we proceed to the electrophysiological assay.
- Use the transfer pipette to place the oocyte in the chamber, begin flowing ND96, and measure *V*_m_, pH_i_, and pH_S_ under basal conditions.
- Monitor pH_S_ as we replace ND96 with either the 5% CO_2_/33 mM HCO_3_^−^ solution (Solution #3, Table 1; as in Figs 2*B* and 3) or the 0.5 mM NH_3_/NH_4_^+^ solution (Solution #5, Table 1; as in Figs 5*B* and 6); all oocytes in the study underwent this control assay.
- Immediately upon completion of the pH_S_ assay, use a transfer pipette to return the oocyte from the chamber to its original well and incubate the oocyte at ∼22°C in ND96. We serially process all oocytes from a 24‐well plate up to this stage (i.e., steps ‘C’ through ‘E’), and then pause. Thus, this second period of incubation in ND96 (step ‘E’) ranges from ∼30 min to ∼3 h.
- Use a transfer pipette to move each oocyte in the 24‐well ND96 plate to a corresponding well in one of three new 24‐well plates. Here, the wells contain one of the following three solutions: (F_1_) 1 mM pCMBS dissolved in ND96 (Solution #7, Table 1) for 30 min incubation; see Figs 3*D–F*, 6*D–F* and 7, 8, 9); (F_2_) 100 µM DIDS dissolved in ND96 (Solution #8, Table 1; 60 min incubation; see Figs 3*G*–*I* or 6*G*–*I*); or (F_3_) ND96 without an added drug (‘sham’).
- Wash the oocyte. Except in a few cases (see indented paragraph below), we wash the oocyte ×3 in a petri dish containing ordinary ND96, as follows. We use a transfer pipette to pick up an oocyte from a 24‐plate (step ‘F’) and release the oocyte into a petri dish containing ND96, quickly pick up and release the oocyte into this wash solution twice more (to remove excess drug, if present). At this point, most oocytes progress immediately through step ‘H’ (below). However, in a minority of experiments (see Fig. 3*J*–*L*), we serially treated oocytes with both pCMBS and DIDS. Here, oocytes previously treated with pCMBS (step ‘F_1_’) we exposed to DIDS, as indicated by steps α_1_→α_2_→α_3_. Conversely, oocytes previously treated with DIDS (step ‘F_2_’) we exposed to pCMBS, as indicated by steps β_1_→β_2_→β_3_. In one experiment without any drugs, we also followed the steps γ_1_→γ_2_→γ_3_ (‘double sham’).

Not illustrated in Fig. 1 is a protocol variant that we employ in a few cases (i.e., Fig. 9 *A* and *B*/right bars) in which we introduce an additional step between ‘F_2_’ and ‘G’. Here, after treating an oocyte with DIDS (step ‘F_2_’), we scavenge unreacted DIDS with albumin as follows. After using a transfer pipette to pick up the oocyte from the DIDS‐containing ND96 solution, we release the oocyte into a Petri dish containing 0.2% albumin in otherwise DIDS‐free ND96 (Solution #9, Table 1), and allow the oocyte to remain in the albumin solution for 20 min. We then wash off the albumin in step ‘G’ by transferring the oocyte to a Petri dish containing ordinary ND96, and pick up and release the oocyte ×3 using a transfer pipette.

- Use a transfer pipette to move the oocyte from the Petri dish (step ‘G’) to an identified well in one of three new 24‐well plates, all containing ND96, in preparation for the next assay.
- Transfer the oocyte back to the chamber and (as in step ‘C’), and again measure the new basal *V*_m_, pH_i_, and pH_S_.
- Monitor (as in step ‘D’), for the second time, the pH_S_ transient as we replace ND96 with the CO_2_/HCO_3_^−^ (or NH_3_/NH_4_^+^) solution.

Thus, oocytes in the top row of Fig. 3 (i.e., Fig. 3*A*, *D*, *G* and *J*) went through one of the following two sequences: Panels A→D or A→J, which requires 12–18 h for an entire group of 24 oocytes in a day's work. Not all oocytes survived the entire protocol (as judged by the colour/integrity of the animal pole).

#### *P*_f_ assays

On the same day that we performed our pH_S_ assays, we also performed *P*_f_ assays on separate cells from the same oocyte batch (i.e., oocytes prepared from the same ovary). We: (1) Use a transfer pipette to remove 6 untreated oocytes of each experimental group—hAQP1, C189S mutant, or hAQP1 FLAG‐tagged—from an identified 6‐well plate containing ND96 (i.e., a total of 18 oocytes). (2) Place the oocytes—as quickly as possible—in a Petri dish containing hypotonic ND96 (Solution #6, Table 1). (3) Use a video camera (described above) to monitor cell swelling at 1 image per second over a period of 60 s; oocytes of each group underwent this control assay. (4) Immediately remove the oocytes from the hypotonic solution to identified wells in another Petri dish containing standard ND96 solution to wash the oocytes ×3 at ∼22°C (described above under electrophysiological assays). (5) Incubate the oocytes at ∼22°C either for (5a) 30 min in ND96 containing 1 mM pCMBS (Solution #7, Table 1), or (5b) 1 h in ND96 containing 100 µM DIDS (Solution #8, Table 1). (6) Wash the oocytes (as described under electrophysiological assays, point ‘5a’). (7) Transfer the oocytes to a Petri dish containing hypotonic, drug‐free ND96 as in ‘2’ above. (8) Monitor swelling as in ‘3’ above.

### Generation of the DIDS antibody

#### Generation of DIDS‐fusion protein

We used two rabbits to produce polyclonal antibodies against DIDS, as described by Garcia and Lodish (1989). Briefly, we reacted 2.4 mM DIDS (catalogue # D3514, Sigma–Aldrich), with 5 mg keyhole limpet hemocyanin (KLH; catalogue # H7017, Sigma–Aldrich) in 500 µL of phosphate‐buffered saline (PBS; i.e., 150 mM NaCl, 10 mM phosphate buffer, pH 7.0) in the dark at 37°C × 40 min. Afterwards, we centrifuged the sample × 30 s in an Eppendorf benchtop centrifuge (catalogue # 5415C, TFS) to remove large insoluble aggregates. After pooling yellow supernatants from several centrifuge tubes, we dialysed against PBS at 4°C overnight in a Slide‐A‐Lyzer dialysis cassette (TFS). The following day, we removed the KLH‐DIDS sample from the cassette; quantified the DIDS by absorbance spectroscopy at 340 nm, assuming a molar extinction coefficient (ε) of 54,000 M^−1^ cm^−1^; aliquoted the dialyzed KLH‐DIDS solution; and immediately used it for immunisation.

#### Processing of rabbit blood samples

For both rabbits, we separately processed each of the three blood samples (see above^5^)—2 lots of ∼30–50 mL plus the terminal sample. We centrifuged the blood for 10 min at 1000 ×g using an Eppendorf bench top centrifuge to isolate the serum, and then evaluated antigenicity on western blots of DIDS‐labelled hAQP1. We purified the antibodies (mainly IgG) from the remainder of the serum using a Protein A column (catalogue # 20,356, TFS), following the manufacturer's instructions; estimated [IgG] by measuring absorbance at 280 nm (assuming ε = 210,000 M^−1^ cm^−1^) using a NanoDrop 2000c spectrophotometer (TFS); separated the material into aliquots corresponding to 1 mg/mL of anti‐DIDS antibody; and stored the aliquots at either –20°C for short‐term storage or –80°C for long‐term storage.

We evaluated the antigenicity using western blots of DIDS‐labelled hAQP1‐WT. For the purposes of this study, the antibodies used for detecting DIDS‐labelled hAQP1‐WT were from one lot (i.e., antibodies purified from the sera of one bleed).

### Biochemical experiments

#### Isolation of oocyte membranes

We used a previously described method (Leduc‐Nadeau et al., 2007), except that, after disrupting oocytes and centrifuging, we removed the supernatant and added fresh buffer to the pellet, repeating the procedure until the supernatant was clear (i.e., devoid of yolk).

#### Purification of oocyte membrane proteins

We resuspended isolated oocyte membranes in Tris‐buffered Saline (TBS, 50 mM Tris HCl, pH 7.4, 150 mM NaCl) + 2% n‐dodecyl‐β‐D‐maltopyranoside (DDM; Sol‐Grade, catalogue #D310S, Anatrace, Maumee, OH), incubated at 4°C × 2h, centrifuged at 16,000 × *g* at 4°C × 30 min, and then collected the supernatant containing the solubilized membrane proteins and diluted it so that [DDM] was ≤ 0.5%.

#### Overexpression of hAQP1‐FLAG in Pichia pastoris

We ligated N‐terminally FLAG‐tagged hAQP1 (M DYKDDDDK ASEFKKKL; FLAG sequence underscored, hAQP1 sequence double underscored) into the pPICZ‐A vector (Invitrogen, Carlsbad, CA), induced protein expression with methanol × 72 h at 26 °C, harvested the yeast by centrifugation, and obtained spheroplasts using zymolyase treatment (60 U/mL in 1 M sorbitol, 1 mM EDTA, 10 mM citrate buffer, pH 5.8) as previously described (Gustin et al., 1998). We then equally divided the spheroplasted cells into two groups (i.e., no DIDS and added 100 µM DIDS), incubated × 1 h at ∼22 °C, and then solubilised overnight at 4°C in TBS + 2% DM, with cOmplete Protease Inhibitor Cocktail (catalogue #11 697 498 001, Roche, Mannheim, Germany). Following membrane solubilisation, we clarified the material by low‐speed centrifugation, mixed with anti‐FLAG resin (catalogue #A2220, Sigma–Aldrich), and eluted proteins from a column using TBS + 0.1% DM + 500 µg/mL FLAG peptide (catalogue #F4799, Sigma–Aldrich) at pH 7.4. In some cases, we subjected FLAG‐purified hAQP1 to size‐exclusion chromatography using a Sephadex G‐75 column.

#### Western blotting of membrane proteins from oocytes or Pichia pastoris

We separated proteins extracted from oocyte membranes by SDS‐PAGE using Tris‐Glycine 4–20% gels (BioRad Laboratories, Hercules, CA) or proteins extracted from *Pichia* membranes using 12% Tris‐Glycine gels; transferred the proteins to polyvinylidene difluoride (PVDF) membranes using an iBlot 7‐Minute Blotting System (TFS); and subsequently blocked the cross‐linked proteins with Tris‐buffered saline with Tween (TBST), comprising 25 mM Tris at pH 7.4, 150 mM NaCl, 0.05% Tween‐20 (catalogue # P9416, Sigma–Aldrich)+5% milk powder × 1 h.

We probed blots from oocyte samples with a polyclonal anti‐AQP1 (catalogue # AQP11‐A, Alpha Diagnostics, San Antonio, USA), and blots from *P. pastoris* samples with monoclonal anti‐FLAG (catalogue # F3165‐2MG, Sigma–Aldrich) or polyclonal anti‐DIDS (made in‐house, see above), applying all primary antibodies at 1:1000 dilution from their stocks in TBST + 5% milk, at 4°C overnight. The next day, we washed the blot ×5 with TBST × 10 min. We detected the primary polyclonal antibodies (i.e., anti‐AQP1 and anti‐DIDS) with an HRP‐conjugated goat anti‐rabbit secondary antibody (catalogue # 1 706 515, BioRad Laboratories), and detected the primary monoclonal antibody (i.e., anti‐FLAG) with an HRP‐conjugated goat anti‐mouse secondary antibody (catalogue # STAR207P, BioRad Laboratories). We applied both secondary antibodies at 1:5000 dilution from their stocks in TBST × 1 h, washed the blot ×5 with TBST × 10 min, developed the immunoblots using ECL Prime Western Blotting Detection Reagent (catalogue # 12 316 992, GE Healthcare Amersham, Piscataway, NJ), acquired images with a Typhoon Trio gel‐documentation system (GE Healthcare), and evaluate band density patterns using Image J software (NIH, Bethesda, MD).

### Statistics

We present data as the mean±S.D. (standard deviation), and define N as the number of different frogs used and n as the number of replicate experiments (i.e., oocytes) for each condition in each dataset. We define *m* as the number of comparisons in each dataset. Statistical analyses were performed using Origin 2024 software. Statistical comparisons amongst means were performed using a one‐way analysis of variance (ANOVA) amongst all groups, followed by Holm–Bonferroni mean comparison (Holm, 1979) to control for type I errors across multiple comparisons, establishing the familywise error rate (FWER) at α = 0.05. In brief, the unadjusted *P*‐values for all *m* comparisons in each dataset are listed from lowest to highest. In the first test, we compare the smallest unadjusted *P*‐value to the first adjusted α value, α/*m*. Upon rejection of the null hypothesis, we compare the second‐smallest *P*‐value to the second adjusted α value, α/(*m*–1), and continue the process accordingly. If the unadjusted *P*‐value is ≥ the adjusted α at any stage, the null hypothesis is accepted, rendering all the subsequent hypotheses in the test group null. We conducted one‐sample *t*‐tests (α = 0.05) to ascertain whether the channel‐dependent (ΔpH_S_*)_CO2_ was significantly different from zero when oocytes were treated with both pCMBS and DIDS. If *P*<0.05, the (ΔpH_S_*)_CO2_ was considered significantly greater than zero. We conducted one‐sample *t*‐tests (α = 0.05) to ascertain whether the channel‐dependent (ΔpH_S_*)_NH3_, was significantly different from zero when oocytes were treated with pCMBS. If *P*<0.05, the (ΔpH_S_*)_CO2_ was considered significantly greater than zero.

## Results

### Surface‐pH measurements for hAQP1‐WT and hAQP1‐C189S

#### pH_S_ measurements for CO_2_ transport

Figure 2*A* is a schematic representation of the reaction and diffusion events that take place as we switch the bath solution—that is, the bulk extracellular fluid—from ND96 to another that contains 5% CO_2_/33 mM HCO_3_^−^ at a constant pH of 7.50. This solution change not only leads to a net influx of CO_2_ that lowers intracellular pH (Roos & Boron, 1981) but also lowers CO_2_ concentration near the outer surface of the cell membrane ([CO_2_]_S_). This decrease in [CO_2_]_S_ (1) creates a CO_2_ gradient from the bECF to the cell surface, which leads to partial CO_2_ replenishment at the cell surface but does not alter pH; and (2) drives the reactions HCO_3_^−^ + H^+^ → H_2_CO_3_ → CO_2_ + H_2_O at the cell surface, which also partially replenishes CO_2_. These reactions cause an alkaline pH_S_ transient, the maximal excursion of which we define as ΔpH_S_ (Endeward et al., 2006; Geyer et al., 2013; Musa‐Aziz, Chen et al., 2009). Our colleagues have used 3D reaction‐diffusion models to simulate these pH_S_ and pH_i_ changes as CO_2_ diffuses into a spherical cell (Calvetti et al., 2020; Musa‐Aziz et al., 2014a, 2014b; Occhipinti et al., 2014; Somersalo et al., 2012).

Figure 2*B* shows a representative pH_S_ recording from an H_2_O‐injected or ‘control’ oocyte during such a solution change (for solution composition, see Table 1). The vertical grey bands in the figure indicate periods during which we withdraw the tip of the extracellular pH microelectrode from its previous position, in which its tip created a small dimple (∼40 µm) in the oocyte surface, to a distance of ∼300 µm for recalibration in the bECF, which we assume to have a pH of precisely 7.50. We describe the recalibration in the legend of Fig. 2.

Figure 2*C* shows a representative western blot of membrane preparations (see Methods) of oocytes injected with H_2_O or cRNA encoding either hAQP1‐WT or hAQP1‐C189S. The lower band(s) at ∼25 kDa presumably represent unglycosylated or core‐glycosylated hAQP1 in the endoplasmic reticulum. The upper bands at ∼37 kDa represent mature‐glycosylated hAQP1, much of which is presumably at the plasma membrane. Assuming that the AQP1 antibody has similar sensitivities for the WT and C189S proteins, we conclude that *Xenopus laevis* oocytes express similar amounts of hAQP1 for both WT and C189S.

Figure 2*D* and *E* show the structures of the two inhibitors that we use in the present study. Unlike HgCl_2_, which reacts with a cysteine residue to form the product R‐S‐Hg‐Cl, p‐chloromercuribenzene sulfonate (Fig. 2*D*) forms the product R‐S‐Hg‐R′, where R′ is the benzenesulfonate moiety of pCMBS. We use pCMBS rather than HgCl_2_ because, in our hands, pCMBS is far less toxic to the cells that we study. The inhibitor 4,4′‐diisothiocyanatostilbene‐2,2′‐disulfonate (Fig. 2*E*) has two isothiocyano groups, both of which can react with the deprotonated form of an amino group (R–NH_3_^+^ → R–NH_2_ + H^+^), presumably the ε‐amino group of lysine, to form an N,N‐disubstituted thiourea R–NH‐CS‐NH‐R′, where this R′ is the remainder of the DIDS molecule. Because DIDS is bivalent, it can potentially crosslink two lysine groups. Note that both pCMBS and DIDS are impermeant (Zhao et al., 2025).

Figure 3 shows a series of 12 representative pH_S_ recordings, the first of which, in Fig. 3*A*, is a replicate of Fig. 2*B*. We can see that the ΔpH_S_ induced by the CO_2_ influx in this H_2_O‐injected control oocyte is much smaller (upward orange arrow in Fig. 3*A*) than in a day‐matched oocyte injected with cRNA encoding hAQP1‐WT (upward green arrow in Fig. 3*B*). For a larger number of similar experiments, Statistics Table 3 shows that the mean ΔpH_S_ induced by the CO_2_ influx into H_2_O‐injected oocytes (as in Fig. 3*A*) is significantly different from the CO_2_‐induced ΔpH_S_ recorded from oocytes expressing hAQP1 (as in Fig. 3*B*).

**Statistics Table 3:** Analysis of the mean pH_S_ transients triggered by a CO_2_/HCO_3_^−^ exposure and subsequent CO_2_ influx from the ‘control’ H_2_O, hAQP1 and hAQP1‐C189S populations presented in Figure 3*A*–*C*. The first four columns display from left to right the cRNA injected, Mean ΔpH_S_ amplitude, standard deviation (S.D.) and number of replicates (*n*) are presented for each type of cRNA injection. The right half of the table presents the one‐way ANOVA with Holm‐Bonferroni post‐hoc means comparisons for each group. FWER α set at 0.05. This half of the table is split into two halves, with the upper‐right half showing the adjusted α‐value for each comparison and the lower‐left half the *P*‐values. Significant *P*‐values are in bold.

					H_2_O	hAQP1‐WT	hAQP1‐C189S
**cRNA**	**ΔpH**_S_	**S.D**.	***n***	*P* \ **α**		**α**	**α**
H_2_O	0.051	0.019	23			0.0167	0.0250
hAQP1‐WT	0.116	0.034	34	***P***	**1.58×10^−12^**		0.0500
hAQP1‐C189S	0.106	0.029	23	***P***	**6.96×10^−9^**	0.194	

**Figure 4:**
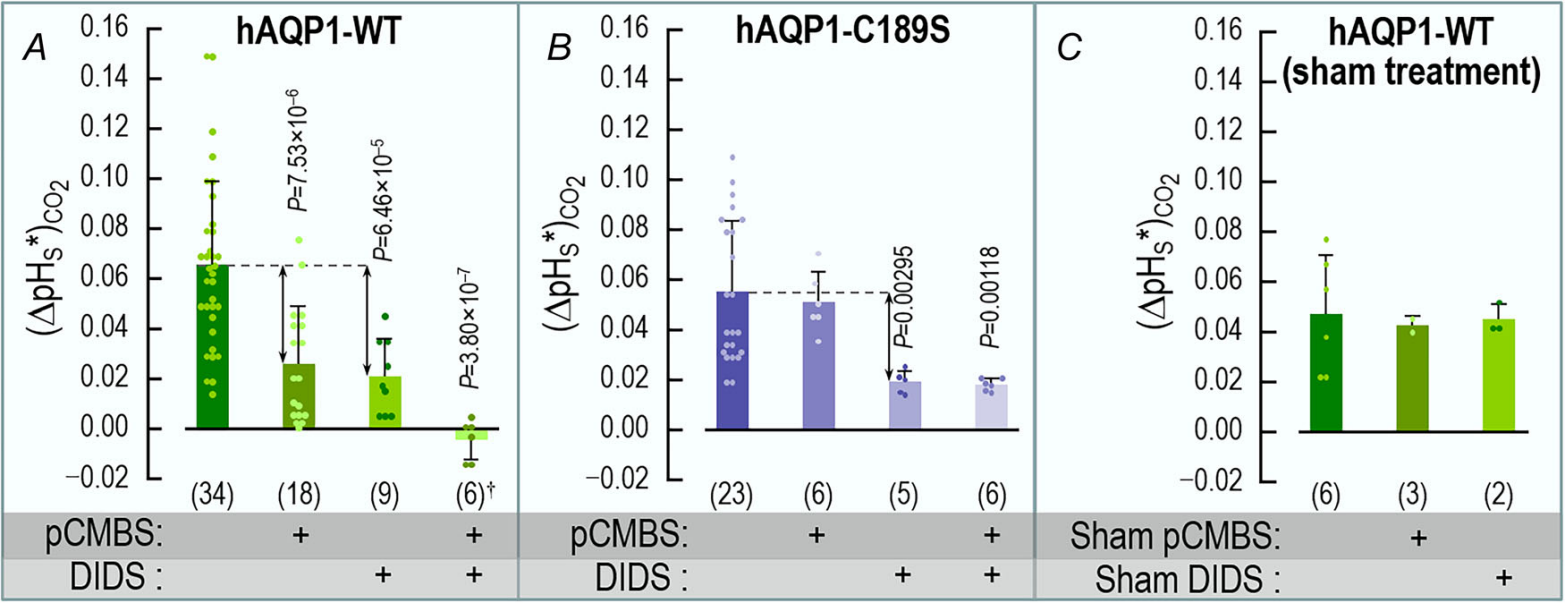
Summary of channel‐specific data in assays for CO_2_ influx A, oocytes expressing hAQP1‐WT. pCMBS and DIDS each reduce (ΔpH_S_*)_CO2_ by somewhat more than half, and the two drugs together reduce it to virtually zero. For the rightmost bar, the dagger symbol (†) adjacent to the replicates (i.e., *n* = 6) indicates that the (ΔpH_S_*)_CO2_ for the pCMBS+DIDS condition is not significantly different from zero (*P* = 0.254, one‐sample *t*‐test). Of the 6 pCMBS+DIDS oocytes, 2 were treated first with pCMBS, and then DIDS; the other 4 were in the opposite order. B, oocytes expressing the C189S mutant of hAQP1. DIDS reduces (ΔpH_S_*)_CO2_ by about half, but pCMBS is without effect, ±DIDS. Of the 6 pCMBS+DIDS oocytes (far‐right bar), 4 we treated first with pCMBS, and then DIDS; the other 2 were in the opposite order. C, oocytes expressing hAQP1 but undergoing only sham drug exposures. These sham experiments show that the long protocols did not have a substantial effect on (ΔpH_S_*)_CO2_. The pCMBS shams (30 min) and DIDS shams (60 min) differed only in the ND96 incubation time in Fig. 1/F_3_. The data come from experiments like those in Fig. 3, in which we exposed oocytes to 5% CO_2_/33 mM HCO_3_^−^ (for protocol, see Methods and Fig. 1). From each ΔpH_S_ of a channel‐expressing oocyte (e.g., Fig. 3*B*, *E*, *H*, *K* or *C*, *F*, *I*, *L*), we subtract the mean, day‐matched ΔpH_S_ for the corresponding H_2_O‐injected oocytes (e.g., Fig. 3*A*, *D*, *G* and *J*) to calculate the channel‐dependent ΔpH_S_ for CO_2_, that is, (ΔpH_S_*)_CO2_. The (ΔpH_S_*)_CO2_ values from individual oocytes are plotted as dots over the green‐shaded bars in panels A and C, and purple‐shaded bars in panel B. At the base of each bar in parentheses is the number of oocytes (*n*), which come from a minimum of 5 batches of oocytes (i.e., different frogs; N ≥ 5). Error bars represent S.D. In the horizontal grey bands at the bottom of each panel, ‘+’ indicates a pre‐incubation with pCMBS or DIDS, or a sham exposure. *P*‐values denote statistically significant differences from the no‐drug condition, and are results of one‐way ANOVAs amongst all groups, followed by Holm–Bonferroni post hoc means comparisons (see Statistics in Methods). For clarity, we display only *P*‐values that indicate statistical significance; we show the *P*‐values for all comparisons in Statistics Table 4A, Statistics Table 4B and Statistics Table 4C.

We define (ΔpH_S_*)_CO2_ as the difference between the CO_2_‐induced ΔpH_S_ value of an oocyte expressing a channel protein (upward black arrow in inset of Fig. 3*B*) and the mean ΔpH_S_ for all day‐matched control oocytes (e.g., Fig. 3*A*). This difference is a semiquantitative index of the channel‐specific CO_2_ flux (Calvetti et al., 2020; Musa‐Aziz, Chen et al., 2009; Somersalo et al., 2012). The leftmost bar in Fig. 4*A* represents the mean (ΔpH_S_*)_CO2_ value for 34 oocytes expressing hAQP1‐WT in the absence of inhibitors.

To assess the importance of the monomeric pore, we examined the effect of pCMBS, which, similar to HgCl_2_ (Preston et al., 1993), covalently modifies Cys‐189 near the extracellular entrance of the monomeric pore and reduces the component of *P*_f_ due to AQP1, which we define as *P*_f_*. Preston et al showed that HgCl_2_ has no effect on *P*_f_ if oocytes express the C189S mutant of AQP1 (Preston et al., 1993). In Fig. 3*C*, we show that a representative ΔpH_S_ induced by the CO_2_ influx into an oocyte expressing hAQP1‐C189S is substantially larger than that from the H_2_O oocyte shown in Fig. 3*A*. For a larger number of similar experiments, Statistics Table 3 shows that the mean ΔpH_S_ of oocytes expressing hAQP1‐C189S is significantly different from that of H_2_O‐injected oocytes but not significantly different from oocytes expressing hAQP1‐WT. On the basis of pH_i_ measurements, Cooper and Boron (1998) showed that pCMBS significantly reduces the CO_2_ permeability of oocytes expressing hAQP1‐WT, but that this effect is absent in oocytes expressing hAQP1‐C189S. In the present study, in which we now monitor pH_S_, experiments on individual oocytes confirm that pretreatment with 1 mM pCMBS (see Methods) reduces the CO_2_‐induced ΔpH_S_ in an hAQP1‐WT oocyte (Fig. 3*B* vs. *E*) but not in an oocyte expressing hAQP1‐C189S (Fig. 3*C* vs. *F*). pCMBS has no effect on an H_2_O‐injected oocyte (Fig. 3*A* vs. *D*), for which ΔpH_S_ is already small. We conclude that pCMBS reduces but does not eliminate the ΔpH_S_ due to hAQP1 (Fig. 3D vs. *E*).

**Statistics Table 4:** Tables of *P*‐values for one‐way ANOVA with Holm‐Bonferroni post‐hoc means comparison for comparisons of differences of channel‐corrected (ΔpH_S_*)_CO2_ on exposure of the oocytes to 5% CO_2_/33 mM HCO_3_^−^. Each table is split into two halves, with FWER α set at 0.05, the upper‐right half shows the adjusted α‐value for each comparison and the lower‐left half the *P*‐value. Significant *P*‐values are highlighted in bold. *4*A*, Statistics summary of channel‐specific data in assays for CO_2_‐influx into hAQP1 expressing oocytes treated with pCMBS, DIDS or pCMBS and DIDS in* Figure *4*A. 4B, Statistics summary of channel‐specific data in assays for CO_2_‐influx into hAQP1‐C189S expressing oocytes treated with pCMBS, DIDS or pCMBS and DIDS in Figure 4B. 4C, Statistics summary of channel‐specific data in assays for CO_2_‐influx into hAQP1 expressing oocytes incubated with sham pCMBS or sham DIDS treatments in Figure 4C

hAQP1‐WT		Ctrl	+pCMBS	+DIDS	+pCMBS & +DIDS
	***P* \ α**		**α**	**α**	**α**
Ctrl			0.0100	0.0125	0.00833
+pCMBS	***P***	**7.53×10^−6^**		0.0500	0.0167
+DIDS	***P***	**6.46×10^−5^**	0.657		0.0250
+pCMBS & +DIDS	***P***	**3.80×10^−7^**	0.0242	0.0902	
hAQP1‐C189S		Ctrl	+pCMBS	+DIDS	+pCMBS & +DIDS
	***P* \ α**		**α**	**α**	**α**
Ctrl			0.0250	0.0100	0.00833
+pCMBS	***P***	0.696		0.0167	0.0125
+DIDS	***P***	**0.00295**	0.0273		0.0500
+pCMBS & +DIDS	***P***	**0.00118**	0.0177	0.946	
hAQP1‐WT		Ctrl	Sham +pCMBS	Sham +DIDS	
	***P* \ α**		**α**	**α**	
Ctrl			0.0167	0.0250	
Sham +pCMBS	***P***	0.768		0.0500	
Sham +DIDS	***P***	0.874	0.887		

The leftmost bar in Fig. 4*B* represents the mean (ΔpH_S_*)_CO2_ from 22 oocytes expressing hAQP1‐C189S in the absence of inhibitors. This (ΔpH_S_*)_CO2_ value is very similar to that for oocytes expressing hAQP1‐WT (leftmost bar in Fig. 4*A*). A comparison of the first and second bars in Fig. 4*A* and *B* shows that pCMBS reduces (ΔpH_S_*)_CO2_ by somewhat more than half in oocytes expressing hAQP1‐WT, but has no significant effect in hAQP1‐C189S oocytes. These data support the hypothesis that, of the CO_2_ that transits hAQP1, a major component moves through the same monomeric pores as H_2_O.

Although studying HCO_3_^−^ transport, Forster et al unexpectedly found that DIDS blocks a large fraction of the CO_2_ permeability of human RBCs (Forster et al., 1998). Endeward et al found that, in RBCs genetically deficient in hAQP1, DIDS has a reduced effect on CO_2_ permeability (Endeward et al., 2006). Here, in the present experiments on single oocytes, we find that pretreatment with 100 µM DIDS reduces the CO_2_‐induced ΔpH_S_ in both an hAQP1‐WT oocyte (Fig. 3*H* vs. *B*) and an hAQP1‐C189S oocyte (Fig. 3*I* vs. *C*), but not in a H_2_O‐injected oocyte (Fig. 3*G* vs. *A*), for which ΔpH_S_ is already small.

The third bars in Fig. 4*A* and *B* summarise mean (ΔpH_S_*)_CO2_ values from a larger group of oocytes pretreated with DIDS. A comparison of the first and third bars shows that DIDS reduces (ΔpH_S_*)_CO2_ by more than half in oocytes expressing either hAQP1‐WT (Fig. 4*A*) or hAQP1‐C189S (Fig. 4*B*). These data are consistent with the hypothesis that a significant fraction of CO_2_ moves through hAQP1 by a DIDS‐sensitive pathway that is unaffected by the C189S mutation in the extracellular mouth of the monomeric pore.

Returning to experiments on individual oocytes, we see that the sequential exposure of an hAQP1‐WT oocyte to pCMBS and then DIDS substantially reduces the CO_2_‐induced ΔpH_S_ (Fig. 3*K* vs. *B*). Moreover, the size of the ΔpH_S_ for the pCMBS/DIDS‐treated hAQP1‐WT oocyte (Fig. 3*K*) is about the same as for H_2_O‐injected oocytes ± inhibitors (Fig. 3*A*,*D*,*G* and *J*). In an hAQP1‐C189S oocyte, the combination of pretreating with pCMBS then DIDS reduces the CO_2_‐induced ΔpH_S_ (Fig. 3*L* vs. *C*), but by no more than DIDS alone (Fig. 3*I* vs. *C*).

The fourth bars in Fig. 4*A* and *B* summarise mean (ΔpH_S_*)_CO2_ values from a larger group of oocytes, in experiments in which we treated with pCMBS and DIDS in either order. A comparison of the first and fourth bars shows that pCMBS+DIDS reduces (ΔpH_S_*)_CO2_ by slightly more than 100% in hAQP1‐WT oocytes (Fig. 4*A*). In the hAQP1‐C189S oocytes, pCMBS+DIDS reduces (ΔpH_S_*)_CO2_ to about the same extent as DIDS alone (Fig. 4*B*), about 40%. Taken together, the data in Figs 3 and 4*A*, *B* are consistent with the hypothesis that, in addition to the component of CO_2_ that moves through the four monomeric pores, another component—at least as large—moves through an entirely separate, DIDS‐sensitive pathway.

Figure 4*C* summarises the results of ND96 sham experiments in which—in step F_3_ of Fig. 1—we simulated either a 30‐min pCMBS exposure (*n* = 3) or a 60‐min DIDS exposure (*n* = 2). The averages of the two sham groups were nearly identical to those of the control group, showing that the oocytes can tolerate these long protocols.

#### pH_S_ measurements for NH_3_ transport

**Figure 5:**
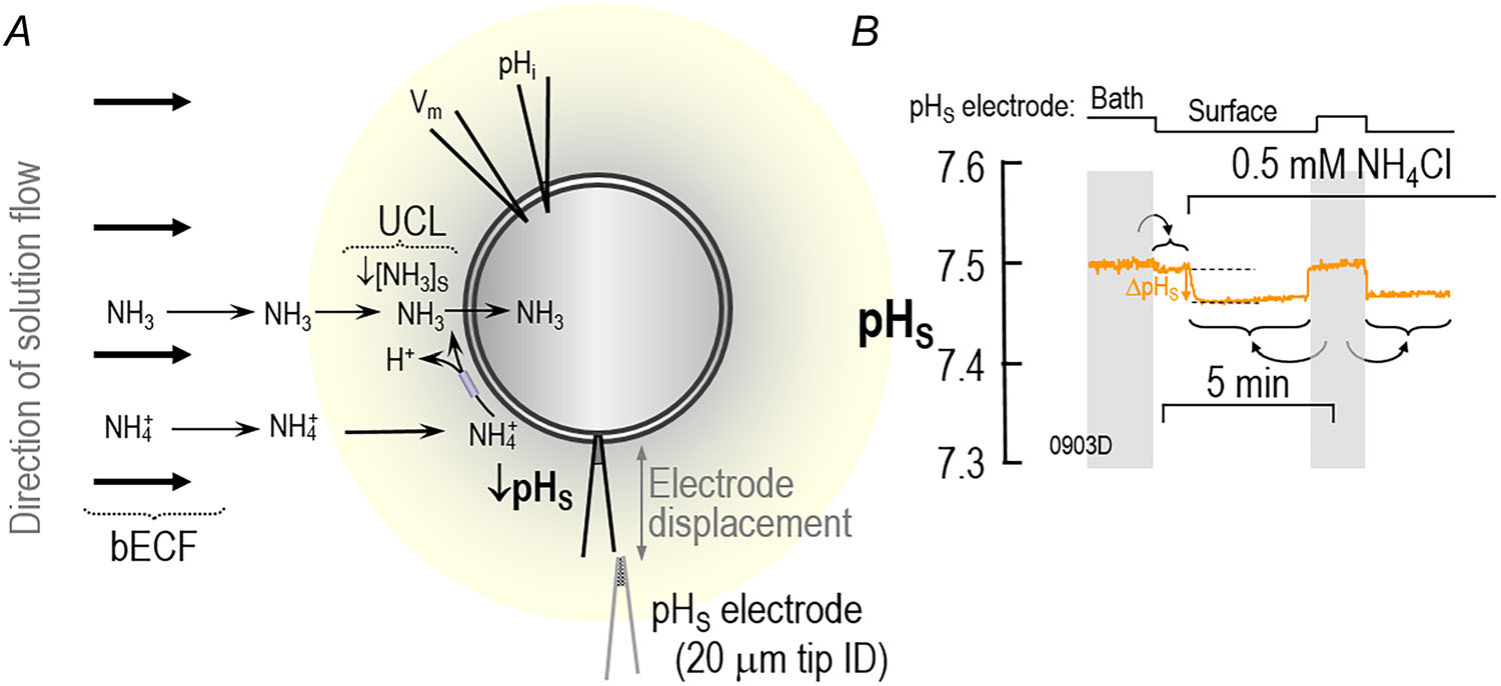
NH_3_ influx into an oocyte. A, schematic illustration of a NH_3_‐influx experiment. Analogous to Fig. 2, the thick black arrows indicate the direction of convective flow within the bulk extracellular fluid (bECF). Thinner arrows indicate solute diffusion or reactions. The maize‐coloured halo indicates the layer of extracellular unconvected fluid (EUF). At the outer surface of the membrane, NH_3_ influx creates an NH_3_ deficit, in part replenished by the reaction NH_4_^+^ → NH_3_ + H^+^ (which decreases pH_S_) and in part replenished by diffusion from the bECF (an isohydric process). The double‐headed arrow indicates movement of the pH_S_ electrode from the bECF to the cell surface for recalibration. B, Example of a surface‐pH (pH_S_) record. The downward arrow indicates the maximal change in pH_S_ (ΔpH_S_) during the application of NH_3_/NH_4_^+^. During the two periods indicated by the vertical grey bands, the tip of the pH_S_ electrode was withdrawn ∼300 µm away from the cell surface for calibration in the bulk extracellular fluid (i.e., pH = 7.50). The curved grey arrows point to horizontal braces that indicate the portions of the experiment to which each calibration pertains. ID, inner diameter. The filename for this representative trace, ‘0903D’—that we reuse in Fig. 6*A*—is annotated at the bottom left corner of the panel.

Figure 5*A* is a schematic representation of the reaction and diffusion events that take place as we expose an oocyte to a solution containing 0.5 mM NH_3_/NH_4_^+^. Here, the changes in pH_S_ are opposite in direction to those caused by CO_2_/HCO_3_^−^ exposure. As the weak base NH_3_ enters the cell, it causes a decrease in [NH_3_]_S_, which has two effects. First, it provides a gradient for NH_3_ diffusion from the bECF to the cell surface. By itself, this diffusion, which partially replenishes NH_3_ at the cell surface, has no effect on pH. Second, the decrease in [NH_3_]_S_ also drives the reaction NH_4_^+^→NH_3_+H^+^ at the cell surface, which also partially replenishes NH_3_. This reaction produces an acidic pH_S_ transient (Musa‐Aziz et al., 2009; Musa‐Aziz, Chen et al., 2009), so that ΔpH_S_<0. Figure 5*B* shows a representative pH_S_ recording as we introduce NH_3_/NH_4_^+^.

**Figure 6:**
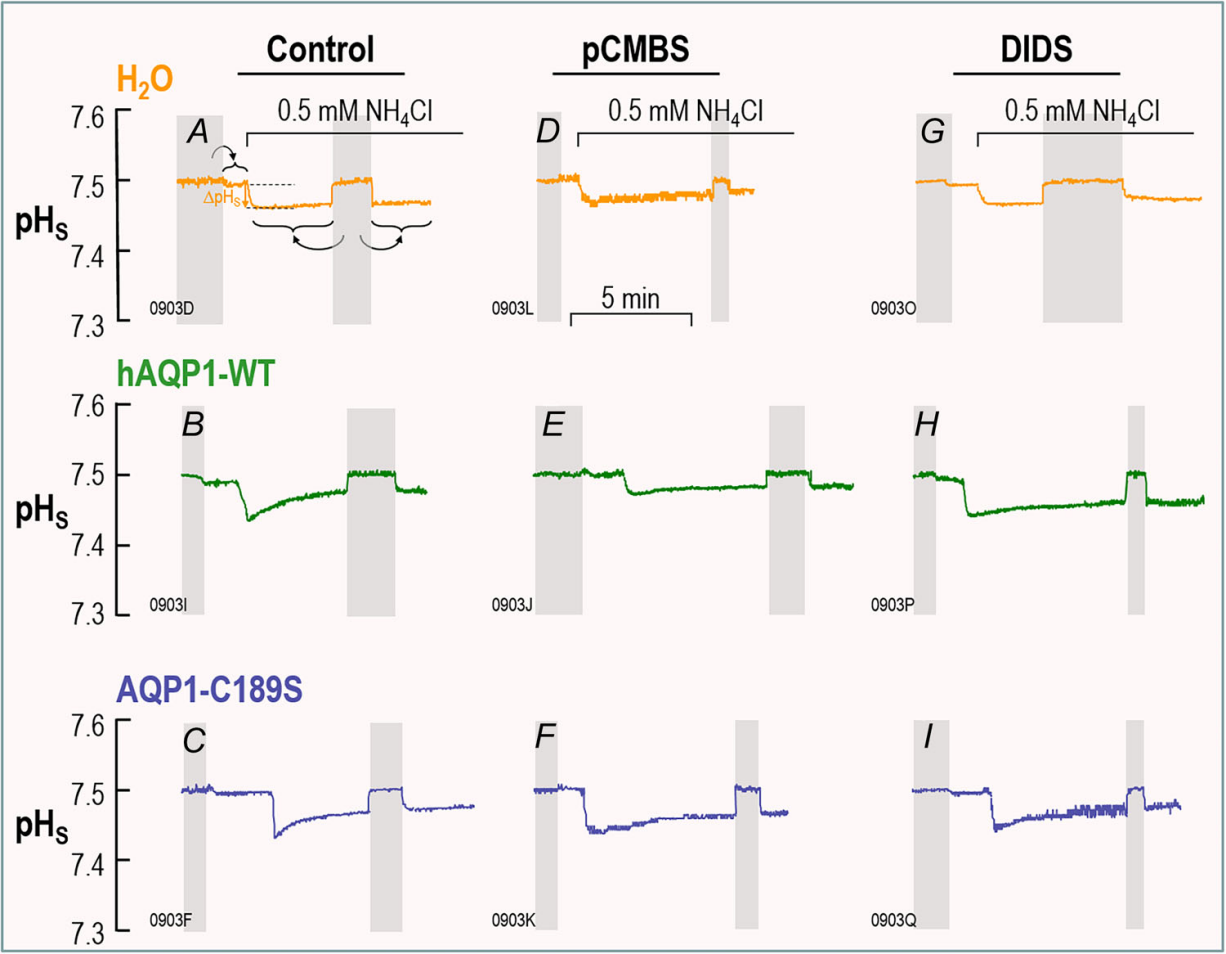
Surface‐pH transients triggered by NH_3_/NH_4_^+^ exposure and the subsequent NH_3_ influx The figure shows representative pH_S_ records from oocytes injected with H_2_O (top row) or cRNA encoding hAQP1‐WT (middle row) or its C189S mutant (bottom row), and later exposed first to the ND96 solution and then, at the indicated times, to a solution containing NH_3_/NH_4_^+^. All oocytes underwent the ‘Control’ protocol, followed by the F_1_ or F_2_ protocol in Fig. 1, although some oocytes did not survive beyond the control protocol (see Methods). Statistics Table 6 presents the analysis of the mean ΔpH_S_ magnitude recorded from all H_2_O, hAQP1 or hAQP1‐C189S ‘control’ oocytes for which we show representative traces in panels *A*, *B* and *C*. As necessary, we pre‐incubated oocytes in 1 mM pCMBS for 30 min, or in 100 µM DIDS for 1b h. Neither drug was present in the bulk solution at the time of the assays. The grey bars indicate when we withdrew the pH‐electrode tip from the oocyte surface to the bulk extracellular solution (pH 7.50) for recalibration. In each panel, the filename of the representative trace is annotated at the bottom left corner of the panel. ‘0903D’ in panel A is a reproduction of the recording in Fig. 5*B*.

**Figure 7:**
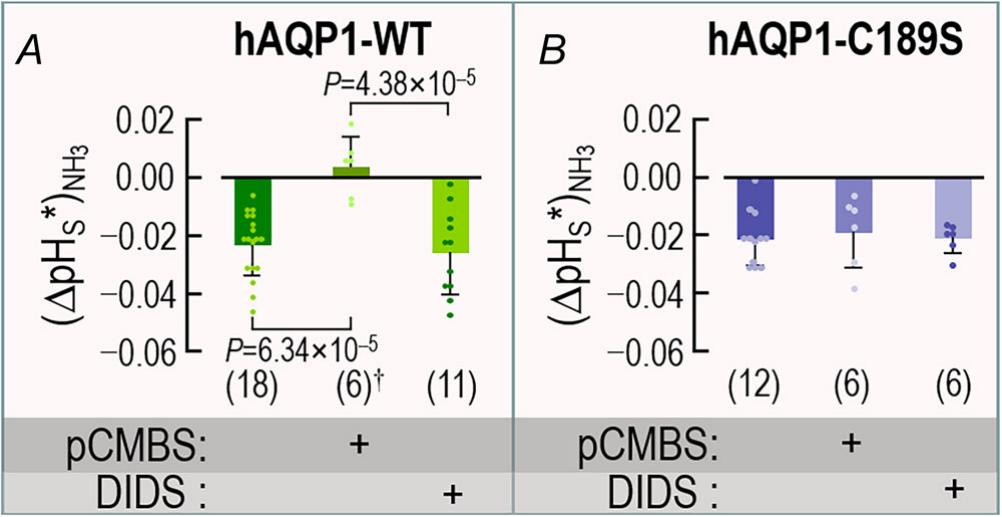
Summary of channel‐specific data in assays for NH_3_ influx A, oocytes expressing hAQP1‐WT. pCMBS reduces (ΔpH_S_*)_NH3_ to virtually zero, whereas DIDS has no effect. For the middle bar, the dagger symbol (†) adjacent to the replicates (i.e., *n* = 6) indicates that the (ΔpH_S_*)_NH3_ for the pCMBS condition is not significantly different from zero (*P* = 0.450, one‐sample *t*‐test). B, oocytes expressing the C189S mutant of hAQP1. Neither drug affects (ΔpH_S_*)_NH3_. The data come from experiments like those in Fig. 6, in which we exposed oocytes to 0.5 mM NH_4_Cl (for protocol, see Methods and Fig. 1). From each ΔpH_S_ of a channel‐expressing oocyte (Fig. 6*B*, *E*, *H* or *C*, *F*, *I*), we subtracted the corresponding mean, day‐matched ΔpH_S_ for H_2_O‐injected oocytes (Fig. 6*A*, *D*, *G*) to calculate the channel‐dependent ΔpH_S_ for NH_3_, that is, (ΔpH_S_*)_NH3_. The (ΔpH_S_*)_NH3_ values from individual oocytes are plotted over the green‐shaded bars in panel A, and purple‐shaded bars in panel B. At the base of each bar in parentheses is the number of oocytes (*n*), which come from a minimum of 5 batches of oocytes (i.e., different frogs; N). Error bars represent S.D. In the horizontal grey bands at the bottom of each panel, ‘+mean ’ indicates a pre‐incubation with pCMBS or DIDS. *P*‐values denote statistically significant differences from the no‐drug condition, and are the results of one‐way ANOVAs amongst all groups, followed by Holm–Bonferroni post‐hoc means comparisons (see Statistics in Methods). For clarity, we display only *P*‐values that indicate statistical significance; we show *P*‐values for all comparisons in Statistics Table 7*A* and Statistics Table 7B.

Figure 6 shows a series of 9 representative pH_S_ recordings on individual oocytes, the first of which, in Fig. 6*A*, is a replicate of Fig. 5*B*. We find that the NH_3_‐induced ‘−ΔpH_S_’ is much smaller in the H_2_O‐injected oocyte (Fig. 6*A*) than in a day‐matched oocyte expressing either hAQP1‐WT (Fig. 6*B*) or hAQP1‐C189S (Fig. 6*C*). For a larger number of similar experiments, Statistics Table 6 shows that the mean ΔpH_S_ induced by NH_3_ influx into H_2_O‐injected oocytes (as in Fig. 6*A*) is significantly different from the NH_3_‐induced ΔpH_S_ recorded from oocytes expressing either hAQP1‐WT (as in Fig. 6*B*) or hAQP1‐C189S (as in Fig. 6*C*); however, the ΔpH_S_ values for oocytes expressing the two hAQP1 constructs were not significantly different. Subtracting the ΔpH_S_ from the H_2_O‐injected oocyte from the ΔpH_S_ from the hAQP1‐WT oocyte yields the channel‐dependent, NH_3_‐induced ΔpH_S_—(ΔpH_S_*)_NH3_ (Musa‐Aziz, Chen et al., 2009). The leftmost bar in Fig. 7*A* represents the mean (ΔpH_S_*)_NH3_ for 18 oocytes expressing hAQP1‐WT in the absence of inhibitors. In experiments on representative oocytes, we find that pretreating an hAQP1‐WT oocyte with 1 mM pCMBS markedly reduces ‘−ΔpH_S_’ (Fig. 6*E* vs. *B*), but has no effect in an hAQP1‐C189S oocyte, where ‘–ΔpH_S_’ remains high (Fig. 6*F* vs. *C*); or in an H_2_O oocyte, where ‘–ΔpH_S_’ remains low (Fig. 6*D* vs. *A*). The leftmost bar in Fig. 7*B* represents the mean (ΔpH_S_*)_NH3_ value from 12 oocytes expressing hAQP1‐C189S in the absence of inhibitors. This (ΔpH_S_*)_NH3_ value is virtually identical to that for oocytes expressing hAQP1‐WT (leftmost bar in Fig. 7*A*). A comparison of the first and second bars show that pCMBS reduces ‘–(ΔpH_S_*)_NH3_’ by virtually 100% in oocytes expressing hAQP1‐WT (Fig. 7*A*), but has no effect in hAQP1‐C189S oocytes (Fig. 7*B*).

**Statistics Table 6:** Analysis of the mean pH_S_ transients triggered by a 0.5 mM NH_4_Cl exposure from the ‘control’ H_2_O, hAQP1‐WT and hAQP1‐C189S populations presented in Figure 6*A*, *B* and *C*. The first four columns display, from left to right, the cRNA injected, the mean ΔpH_S_ amplitude, standard deviation (S.D.), and number of replicates (n) presented for each type of cRNA injection. The right‐half of the table presents the one‐way ANOVA with Holm‐Bonferroni post‐hoc means comparisons for each group. FWER α set at 0.05. This half of the table is split into two halves, with the upper‐right half showing the adjusted α‐value for each comparison and the lower‐left half the *P*‐value. Significant *P*‐values are highlighted bold.

					H_2_O	hAQP1‐WT	hAQP1‐C189S
**cRNA**	**ΔpH**_S_	**S.D**.	***n***	*P* \ **α**		**α**	**α**
H_2_O	0.0387	0.013	25			0.0167	0.0250
hAQP1‐WT	0.0618	0.011	18	***P***	**6.77×10^−8^**		0.0500
hAQP1‐C189S	0.0600	0.009	12	***P***	**4.74×10^−6^**	0.689	

**Statistics Table 7:** Tables of *P*‐values for one‐way ANOVA with Holm‐Bonferroni post‐hoc means comparison for channel corrected (ΔpH_S_*)_NH3_ during exposure of the oocytes to 0.5 mM NH_4_Cl. Each table is split into two halves by the black‐shaded cells, with FWER α set at 0.05, the upper‐right half shows the adjusted α‐value for each comparison and the lower‐left half the *P*‐value. Significant *P*‐values are highlighted bold. 7A, Statistics summary of channel‐specific data in assays for NH_3_‐influx into hAQP1 expressing oocytes treated with pCMBS, or DIDS in Figure 7A. 7B, Statistics summary of channel‐specific data in assays for NH_3_‐influx into hAQP1‐C189S expressing oocytes treated with pCMBS, or DIDS in Figure 7B.

hAQP1‐WT		Ctrl	+pCMBS	+DIDS
	***P* \ α**		**α**	**α**
Ctrl			0.0250	0.0500
+pCMBS	***P***	6.34×10–5		0.0167
+DIDS	***P***	0.551	4.38×10–5	
hAQP1‐C189S		Ctrl	+pCMBS	+DIDS
	***P* \ α**		**α**	**α**
Ctrl			0.0167	0.0500
+pCMBS	***P***	0.636		0.0250
+DIDS	***P***	0.984	0.694	

**Figure 8:**
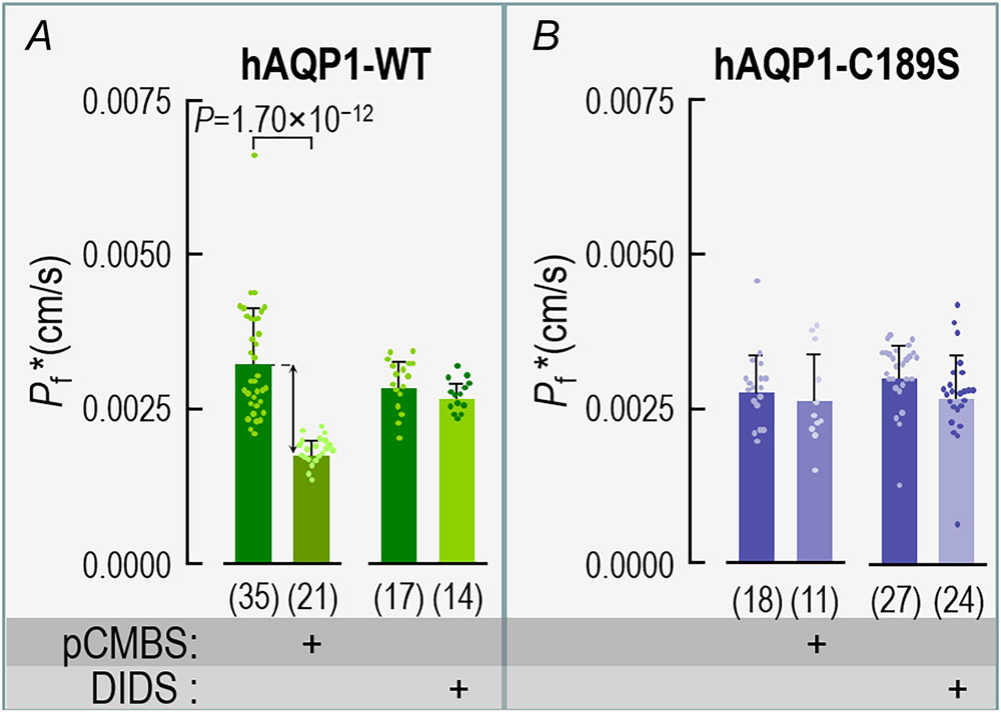
Summary of channel‐specific data in assays for osmotic water permeability A, oocytes expressing hAQP1‐WT. pCMBS but not DIDS reduces *P*_f_. B) oocytes expressing the C189S mutant of hAQP1. Neither drug reduces *P*_f_. From each *P*_f_ from a channel‐expressing oocyte, we subtracted the corresponding mean, day‐matched *P*_f_ for H_2_O‐injected oocytes to calculate the channel‐dependent *P*_f_, that is, *P*_f_*. The *P*_f_* values from individual oocytes are plotted over the green‐shaded bars in panel A, and purple‐shaded bars in panel B. At the base of each bar in parentheses is the number of oocytes (*n*), which come from a minimum of 5 batches of oocytes (i.e., different frogs; N). Error bars represent S.D. In the horizontal grey bands at the bottom of each panel, ‘+’ indicates a pre‐incubation with pCMBS or DIDS. *P*‐values denote statistically significant differences from the no‐drug condition, and are the results of a one‐way ANOVA amongst all groups, followed by Holm–Bonferroni post‐hoc means comparisons (see Statistics in Methods). For clarity, we display only *P*‐values that indicate statistical significance; we show *P*‐values for all comparisons in Statistics Table 8.

**Statistics Table 8:** for Figure 8 Tables of *P*‐values for one‐way ANOVA with Holm‐Bonferroni post‐hoc means comparison for channel corrected *P*_f_^*^ after incubation in no drug control (Ctrl), +pCMBS or +DIDS solutions. Ctrl oocytes are separated into two groups. Those from the same oocyte preparations (frogs) pre‐incubated with pCMBS and those from the same oocyte preparations pre‐incubated with DIDS. Each table is split into two halves by the black‐shaded cells, with FWER α set at 0.05, the upper‐right half shows the adjusted α‐value for each comparison, and the lower‐left half the *P*‐value. Significant *P*‐values are highlighted in bold. Cells shaded grey are comparisons between conditions performed on oocytes from different frogs; therefore, the comparisons are not pertinent even if the *P*‐value is statistically significant. 8A, Statistics summary channel‐specific P_f_ data in assays for osmotic water permeability for hAQP1 oocytes in Figure 8A. 8B, summary channel‐specific P_f_ data in assays for osmotic water permeability for hAQP1‐C189S oocytes in Figure 8B

hAQP1‐WT		Ctrl for +pCMBS	+pCMBS	Ctrl for +DIDS	+DIDS
	***P* \ α**		**α**	**α**	**α**
Ctrl for +pCMBS			0.00833	0.0250	0.0167
+pCMBS	***P***	**1.70×10^−12^**		0.0100	0.0125
Ctrl for +DIDS	***P***	0.0632	**9.02×10^−7^**		0.0500
+DIDS	***P***	**0.00761**	**8.72×10^−5^**	0.395	
hAQP1‐C189S		Ctrl for +pCMBS	+pCMBS	Ctrl for +DIDS	+DIDS
	***P* \ α**		**α**	**α**	**α**
Ctrl for +pCMBS			0.0167	0.0125	0.0500
+pCMBS	***P***	0.514		0.0100	0.0250
Ctrl for +DIDS	***P***	0.171	0.0644		0.00833
+DIDS	***P***	0.722	0.702	0.0619	

**Figure 9:**
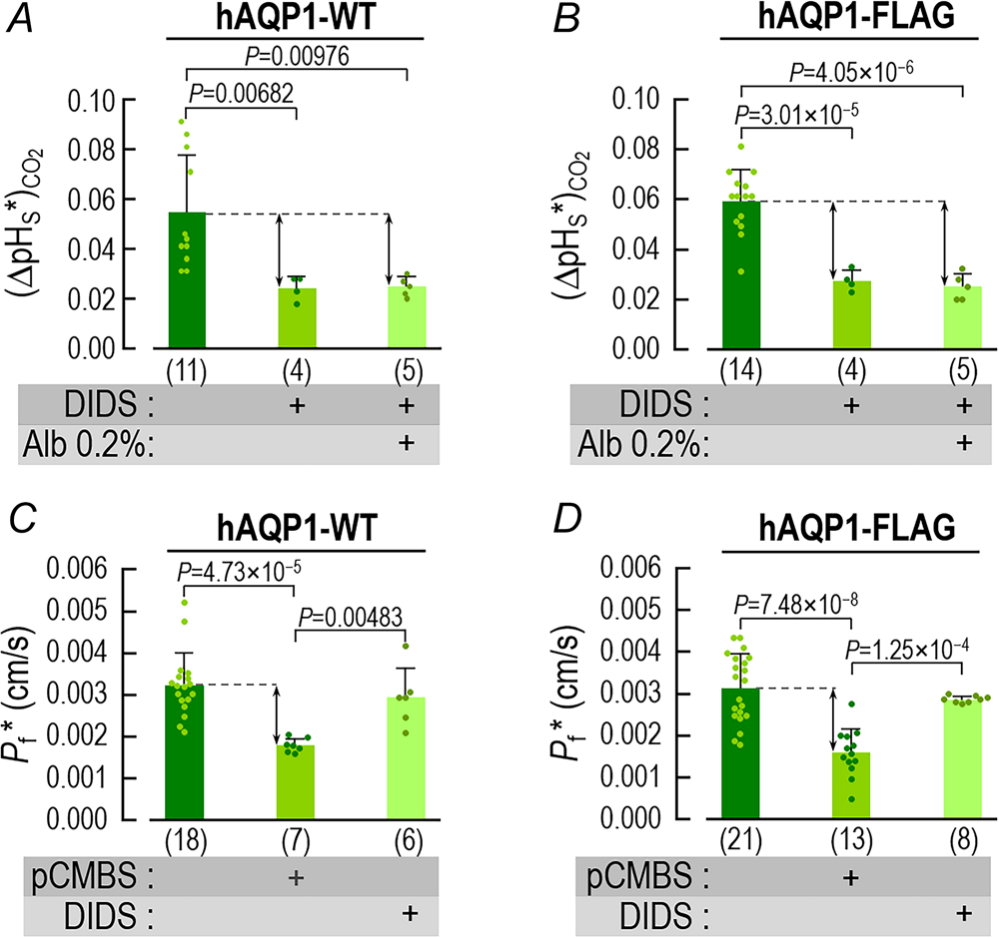
Summary of channel‐specific data for CO_2_ and H_2_O influx for WT versus FLAG‐tagged hAQP1 A, Oocytes expressing hAQP1‐WT: Effect of albumin washes on the DIDS sensitivity of (ΔpH_S_*)_CO2_. DIDS reduces (ΔpH_S_*)_CO2_ by about half, regardless of the subsequent albumin wash. B, Oocytes expressing N‐terminally FLAG‐tagged hAQP1: Effect of albumin washes on the DIDS sensitivity of (ΔpH_S_*)_CO2_. Also, in the FLAG‐tagged construct, DIDS reduces (ΔpH_S_*)_CO2_ by about half, regardless of the albumin wash. C, Oocytes expressing hAQP1‐WT: The DIDS sensitivity of *P*_f_*. These experiments are a matched control for the study in panel D. As in the comparable experiments summarised in Figure 8A, pCMBS but not DIDS reduces *P*_f_. D, Oocytes expressing FLAG‐tagged hAQP1: The DIDS sensitivity of *P*_f_*. As in panel C (no FLAG tag), pCMBS but not DIDS reduces *P*_f_ in oocytes expressing FLAG‐tagged hAQP1. For panels A and B, the control pH_S_ data (leftmost dark‐green bars) come from experiments like those in Figure 3, whereas for panels C and D, the control *P*_f_ data come from experiments like those in Figure 8. The (ΔpH_S_*)_CO2_ or *P*_f_* values from individual oocytes are plotted as dots over the green‐shaded bars in panels A through D. At the base of each bar in parentheses is the number of oocytes (*n*), which come from a minimum of 5 batches of oocytes (i.e., different frogs; N ≥ 5). Error bars represent S.D. In the horizontal grey bands at the bottom of each panel, ‘+’ indicates a pre‐incubation with pCMBS or DIDS, or the presence of 0.2% bovine serum albumin (Alb 0.2%) *P*‐values denote statistically significant differences from the no‐drug condition, and are results of one‐way ANOVAs amongst all groups, followed by Holm–Bonferroni which means comparisons (see Statistics in Methods). For clarity, we display only the *P*‐values that indicate statistical significance; we show *P*‐values for all comparisons in Statistics Table 9.

**Statistics Table 9:** for Figure 9 Tables of *P*‐values for one‐way ANOVA with Holm‐Bonferroni post‐hoc means comparison for channel corrected A, (ΔpH_S_*)_CO2_ in hAQP1‐WT oocytes, B, (ΔpH_S_*)_CO2_ in hAQP1‐FLAG tagged oocytes, C, *P*_f_^*^ in hAQP1‐WT oocytes and D, *P*_f_^*^ in hAQP1‐FLAG tagged oocytes. Each table is split in two halves by the black‐shaded cells, with FWER α set at 0.05, the upper‐right half shows the adjusted α‐value for each comparison and the lower‐left half the *P*‐value. Significant *P*‐values are highlighted bold. 9A, Statistics summary of channel‐specific CO_2_ influx data for into hAQP1‐WT oocytes. 9B, Statistics summary of channel‐specific CO_2_ influx data for into hAQP1‐FLAG oocytes. 9C, Statistics summary of channel‐specific P_f_ data for into oocytes expressing hAQP1‐WT. 9D, Statistics summary of channel‐specific P_f_ data for into hAQP1‐FLAG oocytes.

hAQP1‐WT		Ctrl	+DIDS	+DIDS +Alb 0.2%
	***P* \ α**		**α**	**α**
Ctrl			0.0250	0.0167
+DIDS	***P***	**0.00976**		0.0500
+DIDS +Alb 0.2%	***P***	**0.00682**	0.955	
hAQP1‐FLAG		Ctrl	+DIDS	+DIDS +Alb 0.2%
	***P* \ α**		**α**	**α**
Ctrl			0.0250	0.0167
+DIDS	***P***	**3.01×10^−5^**		0.0500
+DIDS +Alb 0.2%	***P***	**4.05×10^−6^**	0.740	
AQP1		Ctrl	+pCMBS	+DIDS
	***P* \ α**		**α**	**α**
Ctrl			0.0167	0.0500
+pCMBS	***P***	**4.73×10^−5^**		0.0250
+DIDS	***P***	0.361	**0.00483**	
hAQP1‐FLAG		Ctrl	+pCMBS	+DIDS
	***P* \ α**		**α**	**α**
Ctrl			0.0167	0.0500
+pCMBS	***P***	**7.49×10^−8^**		0.0250
+DIDS	***P***	0.322	**1.25×10^−4^**	

Returning to experiments on individual oocytes, we see that DIDS has no effect on ‘−ΔpH_S_’ in an H_2_O‐injected control oocyte (Fig. 6*G* vs. *A*), where ‘–ΔpH_S_’ remains low; or in an hAQP1‐WT oocyte (Fig. 6*H* vs. *B*) or an hAQP1‐C189S oocyte (Fig. 6*I* vs. *C*), where ‘–ΔpH_S_’ remains high. The third bars in Fig. 7*A* and *B* summarise mean (ΔpH_S_*)_NH3_ values from larger groups of DIDS‐pretreated oocytes. A comparison of the first and third bars in each panel shows that DIDS is without effect on (ΔpH_S_*)_NH3_ in oocytes expressing hAQP1‐WT (Fig. 7*A*) or hAQP1‐C189S (Fig. 7*B*). Taken together, the data in Figs 6 and 7 indicate that all of the NH_3_ transiting hAQP1 moves via the same pathway as H_2_O and one of the two major components of CO_2_—namely the four pCMBS‐sensitive monomeric pores. None of the NH_3_ moves via the alternate, DIDS‐sensitive pathway taken by the other major component of CO_2_.

#### Cell‐swelling experiments

In parallel with the CO_2_ and NH_3_ assays (Figs 4 and 7), we determined *P*_f_*. In Fig. 8*A*, we summarise our hAQP1‐WT data, which confirm earlier work on RBCs and oocytes heterologously expressing AQP1, mercurials reduce *P*_f_* by about half (Kabutomori et al., 2018; Macey, 1984; Musa‐Aziz, Chen et al., 2009; Preston et al., 1993; Zeidel et al., 1994) but that DIDS is without effect (Endeward et al., 2006; Macey, 1984). Figure 8*B* confirms that the hAQP1‐C189S mutation renders *P*_f_* insensitive to pCMBS (Cooper & Boron, 1998; Kabutomori et al., 2018), and also shows that the mutation does not affect the lack of DIDS sensitivity.

### Modification of hAQP1 by DIDS

Via rapid and reversible electrostatic interactions, the two sulfonate groups of DIDS can reversibly interact with cationic sites on proteins. Via slower covalent reactions with the –NH_2_ group of lysine or –OH/–SH groups of other amino acids, the two isothiocyanate groups of DIDS can act as a homobifunctional crosslinking reagent (Cabantchik & Rothstein, 1972; Lepke et al., 1976). The pair of sulfonate groups on DIDS endows this divalent anion with low membrane permeability.

#### pH_S_ experiments on oocytes

To assess whether the DIDS inhibition occurs via an electrostatic or covalent interaction, we followed a DIDS exposure with an albumin wash (see Methods) to scavenge non‐covalently bound DIDS. Figure 9*A* shows that the albumin wash does not significantly reduce the degree to which the DIDS pretreatment decreases (ΔpH_S_*)_CO2_. Because the inhibition is irreversible, the interaction of the DIDS with hAQP1 is most likely covalent. To investigate this covalent modification, we performed the biochemistry experiments (presented in the next section) in which we FLAG‐tagged hAQP1 at its N‐terminus, overexpressed the construct in *Pichia pastoris*, and prepared spheroplasts. Here, in control oocyte experiments, we observe that the FLAG tag has no effect on the (ΔpH_S_*)_CO2_ ±DIDS or ±albumin (Fig. 9*A* vs. *B*). We also find that the FLAG tag is without effect in *P*_f_ assays, ±pCMBS or ±DIDS (Fig. 9C vs. *D*).

#### Biochemistry experiments

**Figure 10:**
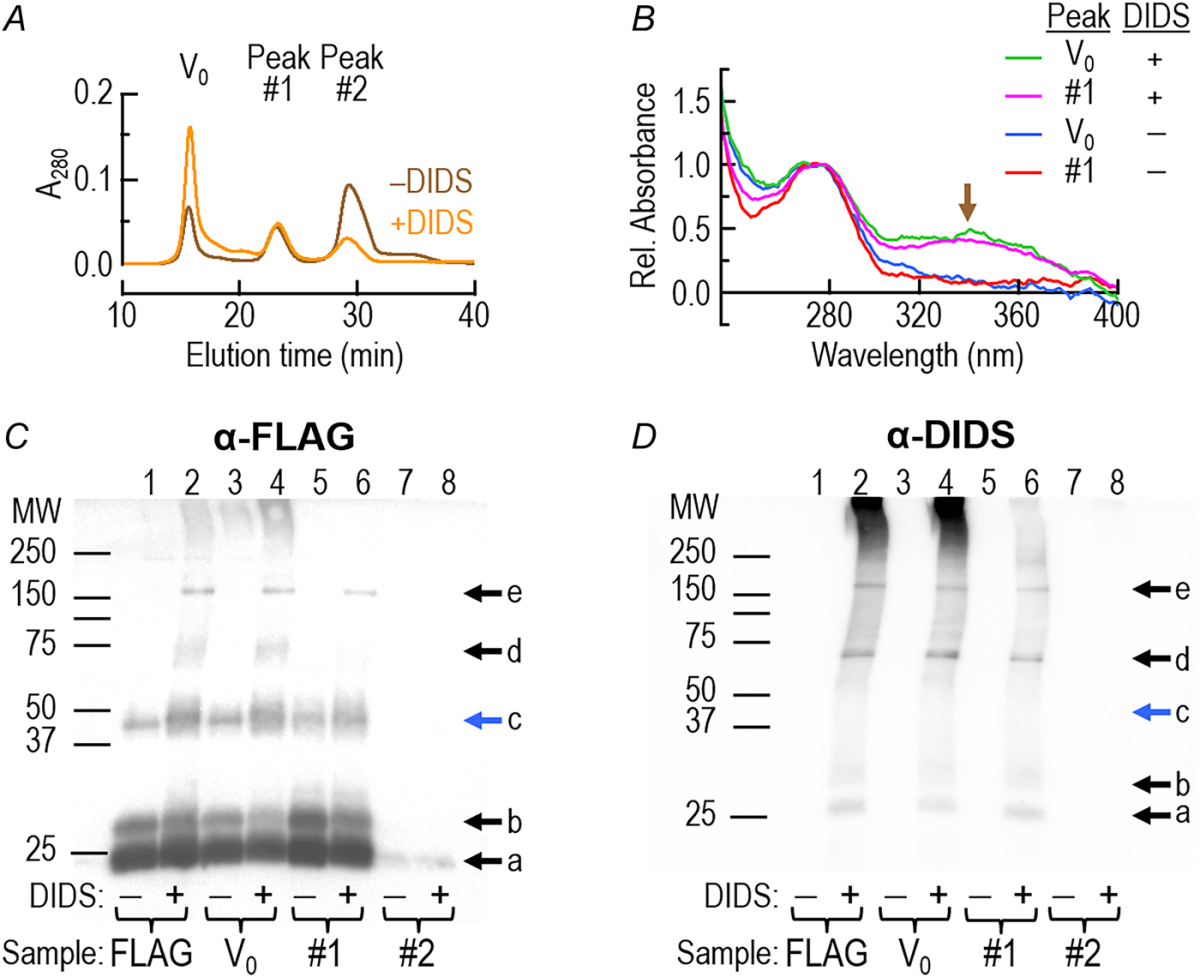
Reaction of DIDS with N‐terminally FLAG‐tagged hAQP1 overexpressed in *Pichia pastoris* A, Size‐exclusion chromatography of solubilised proteins. We treated spheroplasts with DIDS (or no DIDS in parallel, sham experiments), solubilised with DMM, purified N‐terminally FLAG‐tagged hAQP1‐WT using an anti‐FLAG column, and then separated by FPLC on a Superdex‐200 column, recording A_280_ vs. time. The orange record represents a DIDS‐treated sample and the brown record, a control sample (–DIDS) from the same preparation. The first peaks are the void volume (V_0_), which consists of high molecular‐weight (MW) proteins. Peak #1 consists of lower‐MW proteins, and Peak #2, even smaller ones. We translated both records so that A_280_ averaged zero between 10 and 13 min, and then we scaled the orange +DIDS record so that it has the same total area under the curve as Peak #1 in the –DIDS record. B, Absorbance spectra of material (±DIDS) from peaks V_0_ and #1, from a preparation similar to that shown in panel A. We normalised all spectra to unity at 280 nm. The increased absorbance from ∼320 to ∼370 nm in the DIDS‐treated samples presumably represents DIDS, which has an absorbance peak at ∼340 nm (Kodippili et al., 2009). C, Western blot of material from the same experiment as in panel B, probed with anti‐FLAG: Lane 1. Material obtained from spheroplasts not treated with DIDS, and purified on an anti‐FLAG column, as described for panel A, but not subjected to size‐exclusion chromatography. ||| Lane 2. Same as Lane 1, but from DIDS‐treated spheroplasts ||| Lane 3. Peak V_0_ from spheroplasts –DIDS ||| Lane 4. Same as Lane 3, but from spheroplasts +DIDS ||| Lane 5. Peak #1 from spheroplasts –DIDS ||| Lane 6. Same as Lane 5, but from spheroplasts +DIDS ||| Lane 7. Peak #2 from spheroplasts –DIDS ||| Lane 8. Same as Lane 7, but from spheroplasts +DIDS. The presumed assignments are: ‘a’ band <25 kDa, unglycosylated hAQP1 monomer cleaved in the C‐terminus; band ‘b’, 28 kDa, full‐length unglycosylated or core‐glycosylated monomer; band ‘c’, mature glycosylated monomer (blue arrow); band ‘d’ glycosylated dimer; band ‘e’, glycosylated tetramer. D, Western blot of material from the same experiment as depicted in panels B and C, probed with anti‐DIDS. The lane assignments are the same as in panel C. The results in panels A through D are representative of two independent experiments. The absence of i in lanes 1, 3 and 5, especially where there is corresponding immunoreactivity for FLAG‐tagged hAQP1‐WT in panel C, demonstrates the specificity of the anti‐DIDS antibody.

In separate studies, as mentioned in the previous paragraph, we overexpressed FLAG‐tagged hAQP1 in *Pichia pastoris* and prepared spheroplasts. We treated intact spheroplasts with the membrane‐impermeant DIDS (or, as shams, without DIDS), solubilised the membranes in detergent, and purified hAQP1 using an anti‐FLAG resin. Further purification by size‐exclusion chromatography (Fig. 10*A*) shows that DIDS treatment promotes the formation of higher–molecular‐weight hAQP1 species, as indicated by the increased peak height of the void volume (V_0_). We then pooled and concentrated fractions from each peak. Optical spectroscopy of this material (Fig. 10*B*) reveals the expected increase in absorbance centered at ∼340 nm (brown arrow; see Kodippili et al., 2009), due to DIDS in the DIDS‐treated vs. sham samples. We also separated the pooled/concentrated material by SDS‐PAGE and transferred it to membranes for western blotting.

In the anti‐FLAG blot (Fig. 10*C*), the dominant species are hAQP1 monomers (≤50 kDa, arrows a–c). Band a likely represents a C‐terminal cleavage product, and band b probably reflects unglycosylated or core‐glycosylated protein. Thus, both presumably represent mainly intracellular proteins (i.e., not on the plasma membrane). Band c likely represents mature‐glycosylated monomers, some of which may be tetramers that had trafficked to the plasma membrane but dissociated during the preparative procedure. It is perhaps noteworthy that band c is more intense in cells treated with DIDS. Presumed dimers (arrow d) and tetramers (arrow e) are visible only in DIDS‐treated samples (lanes 2, 4, 6), and may represent proteins accessible to DIDS at the outer cell surface. In preliminary work on hAQP1 expressed in oocytes, we similarly found that DIDS markedly increases the appearance of dimers (Geyer et al., 2011).

In the anti‐DIDS blot (Fig. 10*D*), the monomeric species expected to traffic poorly to the plasma membrane (arrows a–b, but dominant in the anti‐FLAG blot) are poorly represented. Indeed, we expect cleaved hAQP1 (band a) and hAQP1 less than mature‐glycosylated (band b) to be largely inaccessible to the impermeant DIDS. Band c, the presumed mature‐glycosylated monomers, is not apparent in the anti‐DIDS blot. We suggest that hAQP1 tetramers at the *Pichia* cell‐surface that did not react with DIDS subsequently dissociated during the preparative process—a possible explanation for the greater intensity of band c in the DIDS‐treated samples in Fig. 10*C* but their virtual absence in Fig. 10*D*. Finally, the presumed hAQP1 dimers and tetramers are clearly visible, though only in DIDS‐treated samples (lanes 2, 4, 6).

These data in Fig. 10 are consistent with the hypothesis that, once the divalent DIDS, with isothiocyano groups at opposite ends of the molecule, reacts to one hAQP1 monomer on the surface of *Pichia* cells, the odds are high that the DIDS crosslinks to one more monomer to yield dimers and tetramers that survive the solubilisation and isolation procedure. Although we attempted to use mass spectrometry to identify hAQP1 residue(s) derivatised by DIDS, we were unable to achieve coverage of protein fragments containing predicted DIDS‐reactive sites near hydrophobic transmembrane segments.

## Discussion

### Pathways for NH_3_ versus CO_2_

#### NH_3_ permeation through monomeric pores

Although previous work had shown that hAQP1 can serve as a conduit for NH_3_ (Nakhoul et al., 2001), the present work is the first direct demonstration that the four monomeric pores of hAQP1—which are responsible for all H_2_O conductance (Preston et al., 1993)—are, in fact, also responsible for all NH_3_ conductance. This result is not unexpected, given the hydrophilicity of the monomeric pore (Sui et al., 2001; Tajkhorshid et al., 2002; Walz et al., 1997), and the similarities of the electronic structures of the NH_3_ molecule (i.e., 1 lone pair of electrons+3 N–H bonds amongst 4 *sp* ^3^ hybrid orbitals) and the H_2_O molecule (i.e., 2 lone pairs+2 O–H bonds amongst 4 *sp* ^3^ hybrid orbitals). Three major lines of evidence support our conclusion that the predominant NH_3_ pathway through hAQP1 is the monomeric pore. First, NH_3_ and H_2_O have similar chemistries, as just noted. Second, pCMBS reduces (ΔpH_S_*)_NH3_ to zero (Fig. 7*A*, centre vs. left bars), an effect abrogated by the C189S mutation (Fig. 7*B*, analogous bars). Third, DIDS has no effect on either (ΔpH_S_*)_NH3_ (Fig. 7*A* and *B*, right vs. left bars) or *P*_f_* (Fig. 8*A*), which leads to a parallel conclusion that virtually none of the NH_3_ or H_2_O moves via the alternate hAQP1 pathway (perhaps the central pore).

Although the above results and interpretations for NH_3_ permeability parallel those for H_2_O permeability, the parallelism is not perfect. As noted in Results^6^, pCMBS reduces (ΔpH_S_*)_NH3_ to zero, but only reduces *P*_f_* by half (Fig. 8). Thus, although the chemistries governing the movements of NH_3_ and H_2_O through the monomeric pore—including the impact of derivatisation of C189 by Hg‐containing compounds—are similar, they are clearly not identical. Finally, as long appreciated from the work of Preston et al. (1993), and confirmed in the present study, derivatisation of C189 does not block all traffic through the monomeric pore of hAQP1.

#### CO_2_ permeation through monomeric pores

Cooper and Boron (1998) had previously examined the effect of pCMBS on the maximal rate of pH_i_ decrease—(dpH_i_/dt)_Max_—elicited by the introduction of 1.5% CO_2_/10 mM HCO_3_^−^. They found that, in oocytes expressing hAQP1‐WT (but not those expressing hAQP1‐C189S or injected with H_2_O), pCMBS causes a change (a decrease) in (dpH_i_/dt)_Max_ that was statistically significant (their Fig. 3*D*). Their statistical analysis did not address the question of whether pCMBS reduced the hAQP1‐dependent component of (dpH_i_/dt)_Max_ to zero^7^. Nevertheless, in hAQP1‐expressing oocytes, they found that pCMBS lowered (dpH_i_/dt)_Max_ nearly to the value observed for H_2_O‐injected control oocytes exposed to pCMBS (their Fig. 3*C*).

The present work shows that, with the same CO_2_/HCO_3_^−^ exposure as Cooper, pCMBS causes a change (a decrease) in (ΔpH_S_*)_CO2_ that is statistically significant; pCMBS causes (ΔpH_S_*)_CO2_ to fall by somewhat more than half. Examination of the Statistics Table 4A shows that, in Fig. 4*A*, the difference between the pCMBS bar and pCMBS+DIDS bar (which we could regard as 100% blockade) falls short of being statistically significant (α = 0.0100, *P* = 0.0242). Thus, from a statistical point of view, the present (ΔpH_S_*)_CO2_ data confirm the earlier (dpH_i_/dt)_Max_ observations of Cooper & Boron (1998). That pCMBS reduces (ΔpH_S_*)_CO2_ by somewhat more than half in the present study but reduced (dpH_i_/dt)_Max_ to nearly background in the Cooper study may reflect differences in the precision of the two methods.

We conclude that at least some CO_2_ moves through hAQP1 via the monomeric pores. Furthermore, on the basis of the present (ΔpH_S_*)_CO2_ data that we regard as being more reliable than the earlier (dpH_i_/dt)_Max_ data, we conclude that the monomeric pathway accounts for about half the total (ΔpH_S_*)_CO2_ signal. Supporting evidence for this conclusion in the present study is that pCMBS reduces (ΔpH_S_*)_CO2_ by somewhat more than half (Fig. 4*A*, second bar from left vs. leftmost bar), an effect abrogated by the C189S mutation (Fig. 4*B*, analogous bars). That some CO_2_ may move through the monomeric pore is not unexpected, given the amphiphilic nature of CO_2_. Moreover, the molecular dynamics simulations of Wang et al. (2007) suggest that some of the CO_2_ permeability of hAQP1 is due to the 4 monomeric pores.

Is it possible that virtually all of the CO_2_ that moves through hAQP1 does so by transiting the monomeric pores? Because treatment with HgCl_2_ or pCMBS blocks only half of *P*_f_*, one might argue by analogy that this pCMBS might block only about half of the CO_2_ traffic through the monomeric pores (i.e., the blocked and unblocked components of CO_2_ traffic through the monomeric pore would sum to ∼100% of all CO_2_ traffic through hAQP1). We believe that this is unlikely for two reasons: (1) CO_2_ is a larger molecule (van der Waals volume ≅ 43 mL/mol) than either NH_3_ (∼37 mL/mol) or H_2_O (∼31 mL/mol). (2) DIDS blocks a major component of (ΔpH_S_*)_CO2_ and yet has no effect on either (ΔpH_S_*)_NH3_ or *P*_f_*. The monomeric‐pore‐only hypothesis for CO_2_ could be true only if DIDS blocks a major component of CO_2_ movement through the monomeric pores and yet does not affect either NH_3_ or H_2_O movement through the monomeric pores, which we believe to be unlikely.

Is it possible that pCMBS does not block all CO_2_ traffic through the monomeric pore? Because (1) pCMBS+DIDS blocks all CO_2_ traffic through hAQP1 and (2) DIDS has no effect on NH_3_ or H_2_O traffic (which occurs exclusively via monomeric pores), the most straightforward explanation for our data is that pCMBS blocks nearly all of the CO_2_ traffic that occurs through monomeric pores.

#### CO_2_ permeation through a parallel pathway

Assuming that DIDS does not block the monomeric pore (see previous sentence), the present work provides the first physiological evidence for a second pathway for CO_2_ through any AQP tetramer. This alternative pathway is apparently parallel to the four monomeric pores. The primary evidence is that (1) DIDS reduces (ΔpH_S_*)_CO2_ by somewhat more than half in hAQP1‐WT (Fig. 4*A*, third bar from the left vs. leftmost bar), (2) the C189S mutation does not affect the DIDS blockade (Fig. 4*B*, analogous bars), and (3) the combination of pCMBS and DIDS reduces (ΔpH_S_*)_CO2_ to zero for oocytes expressing hAQP1‐WT (Fig. 4*A*, rightmost vs. leftmost bars), but stillreduces it by somewhat more than half for oocytes expressing hAQP1‐C189S (Fig. 4*B*, analogous bars).

#### Molecular dynamics

The simulations of Wang et al. (2007) suggest that a major component of the CO_2_ flux through hAQP1 occurs via the hydrophobic central pore that is largely devoid of H_2_O because it is lined by the sidechains of the following residues (from extracellular to intracellular sides): Val‐50, Leu‐54, and Leu‐58 (all contributed by TM2), and Leu‐174 and Leu‐170 (from TM5). The mobility of a gas like CO_2_ through such a near vacuum is ∼10^4^ higher than in liquid water (see Boron, 2010; Rumble, 2022). Thus, even though central pores may comprise only a small fraction of total membrane surface area, it is possible that such pores—on the background of a membrane with a low intrinsic CO_2_ permeability in the absence of AQP1 (Boron et al., 2011)—could make a significant contribution to overall CO_2_ permeability. We propose that the central pore is the anatomic substrate of the DIDS‐sensitive component of CO_2_ permeability of hAQP1‐WT.

### Effect of inhibitors

#### pCMBS

In Results, together with the presentation of Fig. 2*D*, we noted that the reaction of AQP1‐C189 (R‐SH) with HgCl_2_ (as used by Preston et al., 1993) yields R‐S‐Hg‐Cl as opposed to the reaction with pCMBS (Cl‐Hg‐R′), which yields R‐S‐Hg‐R′. Thus, HgCl_2_ treatment yields a product that has steric effects, whereas pCMBS treatment yields a product with even greater steric effects and the electrostatic effects of the sulfonate group. Although the two agents could very well have different inhibitory profiles within the monomeric pore, it is interesting that we found that pCMBS reduces *P*_f_* to about the same extent as previously observed with HgCl_2_ by Preston et al. (1993).

#### DIDS

With its two disulfonate groups, the large DIDS molecule (i.e., DIDS ^=^) is virtually membrane impermeant. In the case of its interactions with AE1 (Cabantchik & Rothstein, 1972) and NBCe1 (Lu & Boron, 2007), the first interaction of DIDS with a target is a reversible ionic interaction (e.g., with a cluster of protonated lysine groups). The higher the affinity of an ionic binding site for the drug, the greater the chance of one of the two –SCN groups of DIDS to be near a lysine residue as it temporarily deprotonates, permitting the covalent reaction as noted in the presentation of Fig. 2*E*. Once one –SCN group reacts with one target lysine residue, the odds of the second – SCN undergoing a similar reaction with a nearby lysine—the crosslinking reaction—rise enormously in what is known as an avidity effect. In the cases of AE1 and NBCe1, both the reversible ionic interactions and the irreversible covalent reactions block transport.

Because its blockade is irreversible with both hAQP1‐WT (Fig. 9*A*) and hAQP1‐FLAG (Fig. 9*B*), the DIDS interaction with hAQP1 expressed in oocytes has probably already advanced to the formation of at least one covalent bond. We propose that DIDS, via a covalent reaction, fully blocks the parallel pathway of nearly all otherwise‐functional hAQP1 tetramers on the oocyte surface. If this parallel pathway is the central pore, then the DIDS could either (1) covalently interact with a single monomer, ±crosslinking, in such a way as to obstruct the central pore but not the monomeric pore or (2) covalently crosslink two monomers to obstruct the central pore. We recognise that DIDS could, in principle, form multiple types of single‐covalent or crosslinking‐covalent bonds within a hAQP1 tetramer, but that not all may produce the physiological blockade of CO_2_ conductance that we observe in the present oocyte studies.

In our work with DIDS‐treated *Pichia* cells, western blots probed with a DIDS antibody (Fig. 10*D*) reveal presumed dimers and tetramers, but virtually no glycosylated monomers (compare Fig. 10*C* vs. *D*, blue arrows). These results imply that, at least in *Pichia*, once one end of a DIDS molecule reacts with one lysine residue on a hAQP1 monomer at the cell surface, the probability is extremely high (avidity effect) that the opposite end cross‐links to a lysine residue on a different monomer.

The likely targets of DIDS on hAQP1 are two extracellular‐facing lysine residues, K36 (on loop A between TM1 and TM2) and K51 (near the beginning of TM2, which lines the extracellular end of the central pore). Because a tetramer has 4 × 2 such lysines, seven unique types of DIDS crosslinking are possible: within a monomer (1 type), between adjacent monomers (3), and between monomers on opposite sides of the central pore (3). Given the length of the DIDS molecule (see Fig. 2*E*), Dr. Ardi Vahidi‐Faridi (personal communication) identifies the three most likely crosslinks as K36‐K51 within the same monomer, K36‐K51 of adjacent monomers, and K51‐K51 of adjacent monomers.

#### Summing the effects of pCMBS and DIDS

The mathematical simulations of Somersalo et al. (2012) suggest that both (ΔpH_S_)_CO2_ and (dpH_i_/dt)_Max_ have similar sigmoidal dependencies on the log_10_ of the membrane permeability to CO_2_ (*P*_M,CO2_). Our physiological data must fall somewhere in the range where (ΔpH_S_)_CO2_ is predicted to rise approximately linearly with log_10_(ΔpH_S_)_CO2_ (see their Fig. 7*B*). If we assume that a H_2_O‐injected oocyte falls at the lower end of this linear range (their dark‐grey point representing *P*_M,CO2_ = [34.2 cm s−^1^]/[5 × 10^4^]), then a doubling of (ΔpH_S_)_CO2_—representing the contribution of hAQP1—would require that we increase *P*_M,CO2_ by ∼5‐fold (their magenta point for *P*_M,CO2_ = [34.2 cm s−^1^]/[1×10^4^]). Starting from this relatively large (ΔpH_S_)_CO2_, reducing the ΔpH_S_ by half—representing the inhibition by DIDS or pCMBS—would require that we decrease *P*_M,CO2_ by nearly 60% (their gold point for *P*_M,CO2_ = [34.2 cm s−^1^]/[2.5×10^4^]), which represents a nearly 75% reduction of the hAQP1‐dependent component of *P*_M,CO2_. If we were to start our analysis further down the sigmoid curve, the predicted inhibition of *P*_AQP,CO2_, by each drug would be somewhat less than 75%, whereas if we had started further up the curve, the predicted inhibition of the Somersalo model would be much higher than ∼75%.

If pCMBS and DIDS each decrease (ΔpH_S_*)_CO2_ by ∼50%—and if we initiate our analysis at the dark‐grey point (Fig. 7*B* of Somersalo et al., 2012)—then each drug must produce a far greater fractional decrease in *P*_AQP,CO2_. Following the logic of the mathematical simulations, the blockade of the monomeric pore by pCMBS should be accompanied by some degree of blockade of the parallel CO_2_ pathway (e.g., central pore). However, the converse is apparently not true. That is, the blockade of the parallel pathway by DIDS seems not to produce significant effects on the four monomeric pores inasmuch as DIDS has no effect on either (ΔpH_S_*)_NH3_ (Fig. 7) or *P*_f_* (Fig. 8). Such an overlapping effect of pCMBS on the monomeric and parallel pathways is not unreasonable inasmuch as the target of pCMBS (i.e., C189) is near the extracellular‐facing NPA motif and the selectivity filter of the monomeric pore, both of which are in close proximity to TM2, which lines the extracellular half of the central pore. Thus, it is possible that derivatization of C189 by pCMBS could lead to shifting or twisting of TM2, thereby reducing gas permeability through the central pore. We propose that (1) pCMBS not only blocks all CO_2_ and NH_3_ traffic through the monomeric pore but also part of the CO_2_ traffic through the parallel pathway, and (2) DIDS blocks (most) CO_2_ traffic through the parallel pathway but no traffic through the monomeric pores. These hypotheses are consistent with the data of the present paper. Given the approximately log‐linear relationship between *P*_M,CO2_ and (ΔpH_S_)_CO2_—and assuming that pCMBS and DIDS each reduce *P*_AQP,CO2_ by ∼75% (see previous paragraph)—we propose that ∼25% of the hAQP1‐dependent CO_2_ traffic occurs through the four monomeric pores (fully blocked by pCMBS but unaffected by DIDS) whereas ∼75% of this CO_2_ traffic occurs through the single central pore (partially blocked by pCMBS but fully blocked by DIDS).

### Molecular basis of gas selectivity

An important unresolved issue is the molecular basis for gas selectivity by AQPs (Geyer et al., 2013; Musa‐Aziz, Chen et al., 2009).

#### CO_2_

The most straightforward interpretation of the data in the present study is that the amphiphilic CO_2_ molecule transits hAQP1 via 2 pathways: the four hydrophilic monomeric pores (presumably a relatively small fraction, ∼25%) and a parallel pathway (e.g., hydrophobic central pore).

#### NH_3_

The most straightforward interpretation of the present data is that all NH_3_ transits the four hydrophilic monomeric pores of hAQP1; that is, none moves via the parallel pathway (e.g., hydrophobic central pore). Considering the broader family of AQPs, we predict that the hydrophobic parallel pathways (e.g., central pores) are never important routes of NH_3_ conductance, but rather that the characteristics of the monomeric pores determine whether a particular AQP has a relatively high permeability to NH_3_ (AQPs 3, 6, 7, 8, 9), or a relatively low NH_3_ permeability (AQPs 2, 4, 5).

We suggest that, for the AQPs known to be permeable to CO_2_ (AQPs 0, 4, 5, 6, 9), CO_2_ transits some combination of monomeric pores and a parallel, more hydrophobic pathway. For the AQPs with limited CO_2_ conductance (AQPs 2, 3, 7, 8), both the monomeric pores and the parallel pathways presumably have less favourable physico‐chemical characteristics. Because more‐hydrophobic molecules like O_2_ and N_2_ are less likely to transit through the monomeric pores, their permeabilities through various AQPs likely depend mainly on the physico‐chemical characteristics of the parallel, hydrophobic pathway. Thus, the gas‐selectivity profile of each AQP likely depends on the physico‐chemical nature of each gas and the unique set of physico‐chemical properties of the (at least 2) potential pathways through the tetramer.

## Concluding remarks

Our work establishes the proof of principle for blocking two pathways for CO_2_ permeation through hAQP1. We suggest that the pCMBS, even though it targets the monomeric pore and reduces CO_2_ permeation via this route, also reduces CO_2_ permeability through the parallel pathway. We hypothesise that the DIDS almost exclusively inhibits the parallel pathway. If it were possible to eliminate the monomeric pathway and make judicious mutations to the hydrophobic alternate pathway, one might be able to design channels with exquisite selectivity amongst gases. Such designer channels could improve industrial gas handling on a micro‐ or nanoscale, provide a simple approach for venting metabolically produced CO_2_ in diving or space flight, provide control of CO_2_ and N_2_ conduction in agriculture, and enable precise O_2_ control for medical therapies, synthetic biology and life‐support systems in underwater or space environments.

## Additional information

## Competing interests

The authors declare that they have no competing interests.

## Author contributions

R.M.‐A. contributed to the conception and design of the research; performed oocyte experiments; analyzed and interpreted the oocyte data; prepared figures; drafted and edited the manuscript. R.R.G. contributed to the conception and design of the research related to oocyte P_f_ experiments as well as Pichia pastoris research; performed all the Pichia pastoris experiments; analyzed and interpreted all results collected with Pichia pastoris; prepared figures; and drafted the manuscript. S‐K.L. validated the cDNA clones for hAQP1 used in the experiments and contributed to the interpretation of pHS experiments. F.J.M. contributed to the design of the research; prepared the figures; analyzed the statistics and interpreted results; drafted and edited the manuscript. W.F.B contributed to the conception and design of the research, and also edited the manuscript. All authors approved the final version of the manuscript and all qualify for authorship, and all those who qualify for authorship are listed.

## Funding

This work was supported by Fundação de Amparo à Pesquisa do Estado de São Paulo, Office of Naval Research, National Institute of Neurological Disorders and Stroke, National Institute of Diabetes and Digestive and Kidney Diseases, National Heart, Lung, and Blood Institute, National Institute of General Medical Sciences and Multidisciplinary University Research Initiative.

## Supporting information


Peer Review History

## Data Availability

All raw data are deposited into the NIH‐supported Zenodo data repository in common and open formats and are accessible via the persistent identifier DOI: 10.5281/zenodo.16966460.

## Footnotes

1 See Methods > Generation of the DIDS antibody

2 In some later experiments, we instead put the oocytes and Ca^2+^‐free solution into 50‐mL Falcon tubes (catalogue # 14‐959‐49A, TFS) and agitated them using a Tube Rotator (Scientific Equipment Products, Baltimore, MD).

3 The apparatus had this dual‐solution arrangement to enable the use of out‐of‐equilibrium CO_2_/HCO_3_^−^ solutions in experiments that are not part of the present study.

4 To obtain *P*_f_ in the proper units (cm s^−1^) in the above equation, the derivation by Zhang et al. (1990) requires that the division of ‘moles of osmotically active particles’ (in the Δ*Osm* term) by ‘moles of water’ (in the V_W_ term) yield a numerical result that is unitless, which of course is impossible. This inconsistency arises during the derivation of the water‐flux equation when one switches from describing the energy of water (i.e., RT) as ‘per mole of water’ per se to the energy of water ‘per mole of osmotically active solute’ during the introduction of the Van't Hoff equation. The introduction of a phantom conversion factor with a value of unity—but with the units ‘(per mole of osmotically active solute)/(per mole of water)’—eliminates the inconsistency.

5 See Methods > Ethical approval and animal procedures > Rabbits

6 See Results > Surface‐pH Measurements … > Cell‐swelling experiments

7 It was later that our laboratory introduced the practice of subtracting the background signal from day‐matched controls to produce the channel‐dependent signal, indicated by the *
